# Identification of New m^6^A Methylation Modification Patterns and Tumor Microenvironment Infiltration Landscape that Predict Clinical Outcomes for Papillary Renal Cell Carcinoma Patients

**DOI:** 10.3389/fcell.2022.818194

**Published:** 2022-03-17

**Authors:** Bin Zheng, Fajuan Cheng, Zhongshun Yao, Yiming Zhang, Zixiang Cong, Jianwei Wang, Zhihong Niu, Wei He

**Affiliations:** ^1^ Department of Urology, Shandong Provincial Hospital Affiliated to Shandong University, Jinan, China; ^2^ Department of Urology, Shandong Provincial Hospital Affiliated to Shandong First Medical University, Jinan, China; ^3^ Cheeloo College of Medicine, Shandong University, Jinan, China; ^4^ Department of Nephrology, Shandong Provincial Hospital Affiliated to Shandong University, Jinan, China; ^5^ Department of Nephrology, Shandong Provincial Hospital Affiliated to Shandong First Medical University, Jinan, China; ^6^ Department of Urology, Shandong Provincial ENT Hospital Affiliated to Shandong University, Jinan, China

**Keywords:** m 6 A, tumor microenvironment, immunotherapy, mutation burden, survival

## Abstract

N6-methyladenosine (m^6^A) is the product of the most prevalent mRNA modification in eukaryotic cells. Accumulating evidence shows that tumor microenvironment (TME) plays a pivotal role in tumor development. However, the underlying relationship between m^6^A modification and the TME of a papillary renal cell carcinoma (PRCC) is still unclear. To investigate the relationship between m^6^A modification and prognosis and immunotherapeutic efficacy for PRCC, we looked for distinct m^6^A modification patterns based on 23 m^6^A-related genes. Next, the correlation between m^6^A modification patterns and TME-related characteristics was investigated. Then, the intersected differentially expressed genes were selected and the scoring system, denoted as m^6^A score, was established to evaluate m^6^A modification, prognosis, and immunotherapeutic efficacy. In this study, three distinct m^6^A expression clusters were identified. Based on the results of immune cell infiltration analysis and functional analysis, carcinogenic pathways, TME-related immune cells, and pathways were identified as well. More importantly, the established m^6^A score showed good value in predicting clinical outcomes according to results using external cohorts. Specifically, PRCC patients with low m^6^A score value showed better survival, immunotherapeutic response, and higher tumor mutation burden. Furthermore, immunohistochemistry using PRCC clinical samples from our medical center was carried out and verified our results. In conclusion, this study highlights the underlying correlation between m^6^A modification and the immune landscape and, hence, enhances our understanding of the TME and improved the therapeutic outlook for PRCC patients.

## Introduction

Kidney cancer is a heterogenous disease for which several subtypes with different genetic and morphologic characteristics are identified. Renal cell carcinoma (RCC) accounts for the vast majority of histological types of kidney cancer with clear cell renal cell carcinoma (ccRCC) making up 70%–80% and papillary renal cell carcinoma (PRCC) 15%–20% of RCCs (Linehan et al., 2016; Barata and Rini, 2017; Vuong et al., 2019). Although most cases of PRCC are indolent with limited risk of mortality, the overall prognosis for PRCC remains limited (Steffens et al., 19902012).

The tumor microenvironment TME is a cellular environment in which tumor cells and other nonmalignant cells exist, and it is composed of various immune cells and related materials, including lymphocytes, fibroblasts, stromal cells, blood vessels, and so on (Wu and Dai, 2017). The TME acts as the soil of tumor cells, and the great impact of TME on tumorigenesis and tumor immunotherapy has become increasingly evident (Li et al., 2021). In an abnormal TME, immune cells become significantly remodeled, which affects their normal functions, such as proliferation, migration, and differentiation (Binnewies et al., 2018). Therefore, immunosuppression is the essential characteristic of TME. Currently, RCC tumors are considered to be immunogenic, and many studies find that various immune cells could infiltrate into RCC TMEs. However, these immune cells block the effective antitumor responses. Owing to the immunosuppressed state of RCC tumors and the immune-tolerance of TMEs, the response of RCC to immune checkpoint inhibitors (ICIs) is unsatisfactory (Syn et al., 2017).

Due to the advances in RNA sequencing, N6-methyladenosine (m^6^A), the product of the most common type of mRNA modification in eukaryotic cells, has garnered great interest (Qi et al., 2016; Ke et al., 2017). The m^6^A modification is regulated by three types of molecules, known as “writer,” “eraser,” and “reader” molecules (Yang et al., 2018). It is reported that m^6^A modification plays multifaceted roles in tumor development and metastasis (Xiao et al., 2018). Various research investigation indicates that abnormal m^6^A modification occurs in most immune cells, including dendritic cells, regulatory T cells, macrophages, CD4^+^ T cells, and CD8^+^ T cells, and results in tumor escape or immune disorder (Chen et al., 2018; Han et al., 2019; Li et al., 2021). However, it is still unclear whether m^6^A modification in diverse immune cells in the TME is responsible for tumor progression and the effectiveness of ICIs. Therefore, it is essential to determine the potential effects of m^6^A modification on the TME and to explore its clinic value as a new therapeutic tool for treatment of PRCC.

## Materials and Methods

### Data Collection and Processing

The expression data and clinical information for kidney renal papillary cell carcinoma (KIRP) were downloaded directly from the Cancer Genome Atlas (TCGA) (https://cancergenome.nih.gov/), Gene Expression Omnibus (https://www.ncbi.nlm.nih.gov/geo/), and the Cancer Immunome Atlas (TCIA) (https://tcia.at/home). Specific data from 289 KIRP patients and 32 tumor-free patients were obtained from these databases. Copy number variation (CNV) and somatic mutation data were downloaded from TCGA as well. Samples without survival data were removed. The “limma” package was used to normalize gene expression data and transform fragments per kilobase per million (FPKM) values to transcripts per kilobase per million (TPM) value. R (R version 4.0.1) was used to extract and analyze expression data and clinical information. After conducting a comprehensive literature review (Zhang et al., 2020; Gu et al., 2021; Zhong et al., 2021), we identified 23 m^6^A regulators, including *METTL3*, *METTL14*, *METTL16*, *WTAP*, *VIRMA*, *ZC3H13*, *RBM15*, *RBM15B*, *YTHDC1*, *YTHDC2*, *YTHDF1*, *YTHDF2*, *YTHDF3*, *HNRNPC*, *FMR1*, *LRPPRC*, *HNRNPA2B1*, *IGFBP1*, *IGFBP2*, *IGFBP3*, *RBMX*, *FTO*, and *ALKBH5*, representing m^6^A writers, readers, and erasers.

### Identification of Differentially Expressed Genes and Functional Analysis

The “limma” and “ggplot2” packages were used to assess and visualize the differentially expressed genes (DEGs) in KIRP samples and nontumor tissues. Difference with adjust *p* < .01 were considered to be significant. Gene Ontology (GO) and Kyoto Encyclopedia of Genes and Genomes (KEGG) analyses were performed through the “clusterProfiler” package. To determine the differences in biological processes between various m^6^A expression clusters, specifically to estimate the variation in biological processes, gene set variation analysis (GSVA) was conducted by using the “GSVA” package (Hänzelmann et al., 2013). We utilized the gene set “c2.cp.kegg.v6.2-symbols” from the MSigDB database (Liberzon et al., 2011). Here, adjusted *p* < .05 was considered as the threshold.

### Estimation of TME Immune Cell Infiltration and Tumor Mutation Burden

Single-sample gene-set enrichment analysis (ssGSEA) was used to quantify the level of immune infiltration into the PRCC TME (Barbie et al., 2009; Charoentong et al., 2017). The relevant gene set, which marks various TME-infiltrated immune cell subtypes, was collected from previous studies (Barbie et al., 2009; Charoentong et al., 2017). The ssGSEA scores represented the enrichment of different immune cell subtypes in each sample. Tumor mutation burden (TMB) was analyzed with the KIRP somatic mutation data by using the “maftools” R package (Chen and Mellman, 2017). Two TMB sets (high and low TMB) were constructed by using an optimal cutoff value of TMB. We evaluated the difference between the m6Ascore values of two TMB sets.

### Unsupervised Clustering and the Construction of an m^6^A Regulators Model

Owing to relatively small sizes of the KIRP data sets in the Gene-Expression Omnibus (GEO) database, we used the GSE2748 cohort and TCGA KIRP data set to perform unsupervised clustering analysis with the “ConsensusClusterPlus” package (Wilkerson and Hayes, 2010). Here, 1000 repetitions were performed. The expression data of 23 m^6^A genes were extracted from GSE2748. The clustering analysis was performed to classify the KIRP samples into distinct m^6^A expression clusters based on the expression of 23 m^6^A regulators.

To quantify the m^6^A expression cluster of each KIRP sample, the m^6^A score was applied and established as follows. First, we identified intersected DEGs from the constructed m^6^A expression clusters. All KIRP patients were divided into diverse groups *via* unsupervised clustering analysis. Then, the univariate Cox regression analysis was utilized to assess the prognosis of each selected gene. *p* < .05 was considered as the significance criterion. After extracting the prognosis-related regulators, we applied principal component analysis to establish the m^6^A gene model, and the principal components 1 and 2 were selected as signature scores. Finally, m^6^A score was calculated using following formula: m^6^A score = Σ (PC1_i_ + PC2_i_) (where i is the expression of the selected m^6^A related DEGs from the m^6^A expression cluster) (Sotiriou et al., 2006; Zeng et al., 2019).

### Genomic and Clinical Data for ICI Therapy

Then, we investigated whether the established m^6^A expression cluster could predict the response of PRCC to ICI therapy based on two immunotherapy cohorts. After a comprehensive search for gene expression data and complete clinical information of patients treated with ICIs, we finally included two related cohorts. The first cohort involved metastatic melanoma patients treated with the anti-PD-1 drug (pembrolizumab) from the GEO database (GSE78220). Moreover, genomic and clinical data for mTOR inhibitor (everolimus) therapy was downloaded from the Supplementary File appended to published study (Barbie et al., 2009; Charoentong et al., 2017). All raw expression data were normalized using the “limma” package and transformed into the more comparable TPM value.

### Immunohistochemistry

Five pairs of PRCC and adjacent normal tissues were collected from May 2021 to October 2021 from Shandong Provincial Hospital affiliated with Shandong First University. The study was approved by the Ethics Committee of Shandong Provincial Hospital (Approval No. SWYX: NO. 2021-491). IHC was performed according to published method (Wang et al., 2020). All samples were incubated with rabbit polyclonal anti-CD8 (ab101500), anti-CD69 (ab233396), anti-CD163 (ab182422), anti-YTHDF1 (ab252346), anti-YTHDF2 (ab220163), anti-YTHDF3 (ab220161), anti-ZC3H13 (IHC0104123), anti-HNRNPA2B1 (ab31645), and anti-IGFBP2 (ab188200) antibodies overnight at 4°C and then washed. Two pathologists independently assessed the IHC slides.

### Statistical Analysis

The Kruskal–Wallis test was used to estimate the significance of differences between values of three or more groups. Spearman’s correlation analysis was applied to calculate the correlation coefficient between number of TME-infiltrated immune cells and the expression level of m^6^A regulators. We employed the “survminer” package to determine the optimal cutoff value. Based on the optimal cutoff point, all PRCC patients were grouped into high or low m^6^A score sets. Then, the Kaplan–Meier analysis with a log-rank test was conducted to test the prognosis of patients. The mutation landscape of KIRP cohorts was depicted by using the “maftools” package (Mayakonda et al., 2018). Statistical analysis was performed with R packages (version 4.0.1). A two-tailed *p* < .05 was considered to be significant.

## Results

### Genetic Variation and Clinical Relevance of m^6^A Genes in PRCC

**FIGURE 1:**
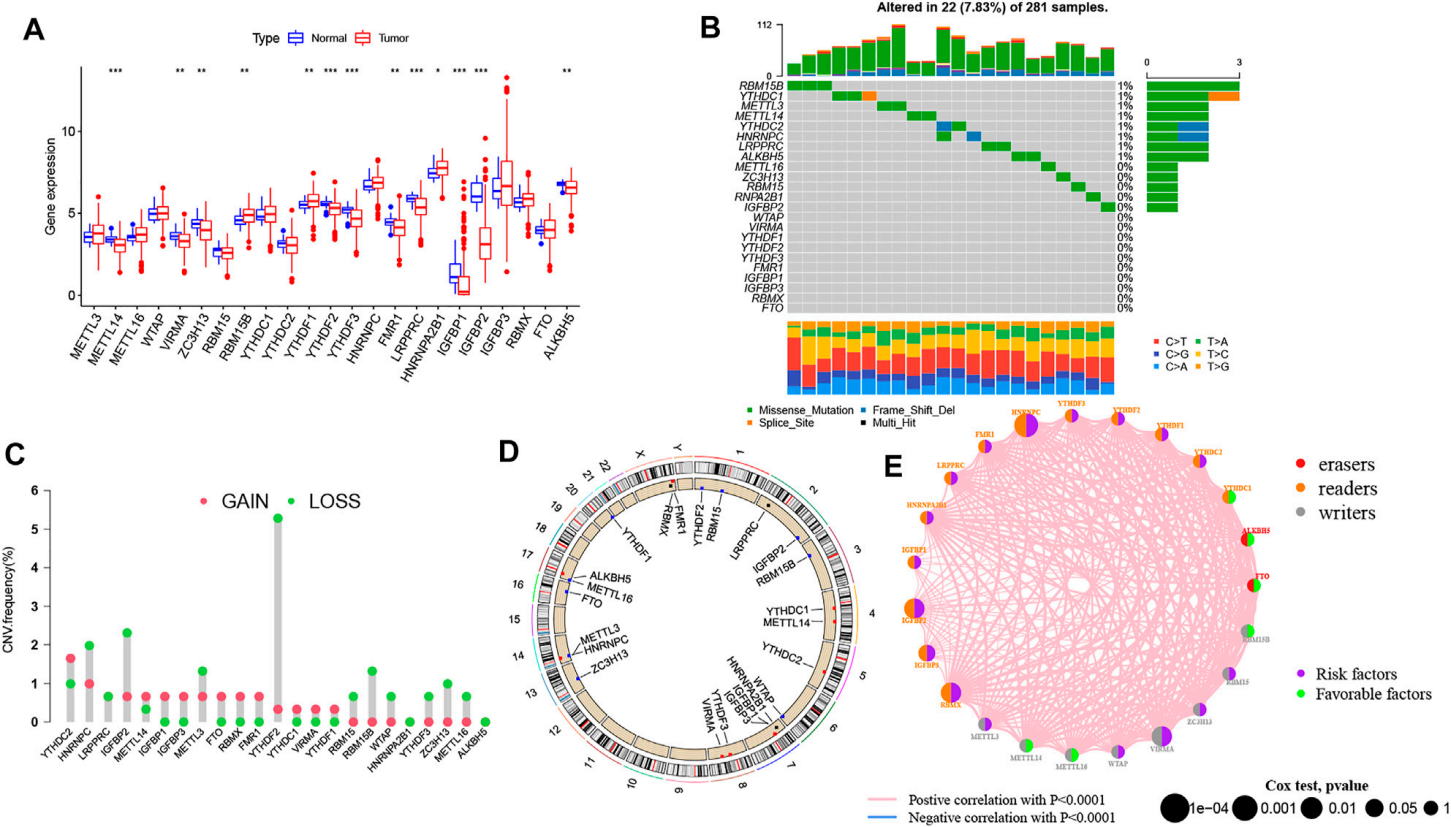
Characteristics of genetic and variation of m^6^A genes in pRCC. **(A)** Transcriptomic profile of 23 m6A genes among normal and PRCC tumor tissues. The upper and lower ends of the boxes indicate interquartile range (IQR) of values. The lines in the boxes represent each the median value. The asterisks represented the *p* value. (*p < .05; **p < .01; ***p < .001) **(B)** Mutation frequency of 23 m^6^A genes in in 289 PRCC patients. The bar plot at the upper part of the figure shows TMB. Numbers on the right part of the figure represent the mutation frequency. **(C)** CNV alteration frequency of 23 m^6^A genes in the 289-patient PRCC cohort. The height of each column indicates the alteration frequency. The red dot represents the amplification frequency; the green dot represents the deletion frequency. **(D)** Location of CNV alterations of 23 m^6^A genes on human chromosomes in PRCC cohort. **(E)** Interaction network of 23 m^6^A genes in the PRCC cohort. The size of each circle is indicative of the magnitude of the effect on the prognosis. The green dots mean the protective factors, and the purple dots represent the risk factors. The thickness of each line indicates the degree of correlation between each gene.

Based on the transcriptomic profiles of 23 m^6^A regulators, we investigated the expression pattern of all m^6^A regulators in PRCC and normal samples from TCGA (Figure 1A). Then, we integrated CNV as well as somatic mutations and illustrated the prevalence of alteration of m^6^A genes in PRCC. Only 22 of 281 samples (7.83%) showed m^6^A regulator mutations. Specifically, 8 out of 23 m^6^A regulators experienced mutations (Figure 1B). Afterward, we investigated the CNV frequency of 23 m^6^A genes, which identified that most CNV alterations in 23 genes were focused on the CNV deletion (Figure 1C). Moreover, we determined the locations of the CNV alteration on human chromosomes as well (Figure 1D). These results indicate that genetic variation commonly occurs in PRCC cells and is heterogeneous between PRCC and normal tissues, exhibiting the potential role for the aberrant expression of m^6^A genes in tumorigenesis and development as well as progression. Finally, when investigating the potential clinical relevance of 23 m^6^A regulators, we found that three types of m^6^A regulators were positively correlated with patient prognosis and interacted with each other (Figure 1E and Supplementary Figure S1A). In addition, most of the genes were indicated to be risk factors for overall survival (OS) of PRCC patients; only *YTHDC1*, *ALKBH5*, *FTO*, *RBM15B*, *METTL14*, and *METTL16* were out.

We also determined whether genetic variations of “writer,” “reader,” and “eraser” genes were associated with the expression other m^6^A regulators’ (Supplementary Figures S1B–L). The results demonstrate that only *YTHDF1* was upregulated in *METTL14* mutated PRCC samples while other m^6^A genes highly expressed in wild-type *ALKBH5*, *HNRNPC*, *METTL14*, *YTHDC1*, and *YTHDC2*.

### Different m^6^A Modification Patterns Mediated by 23 m^6^A Genes and Its Clinical Relevance

**FIGURE 2:**
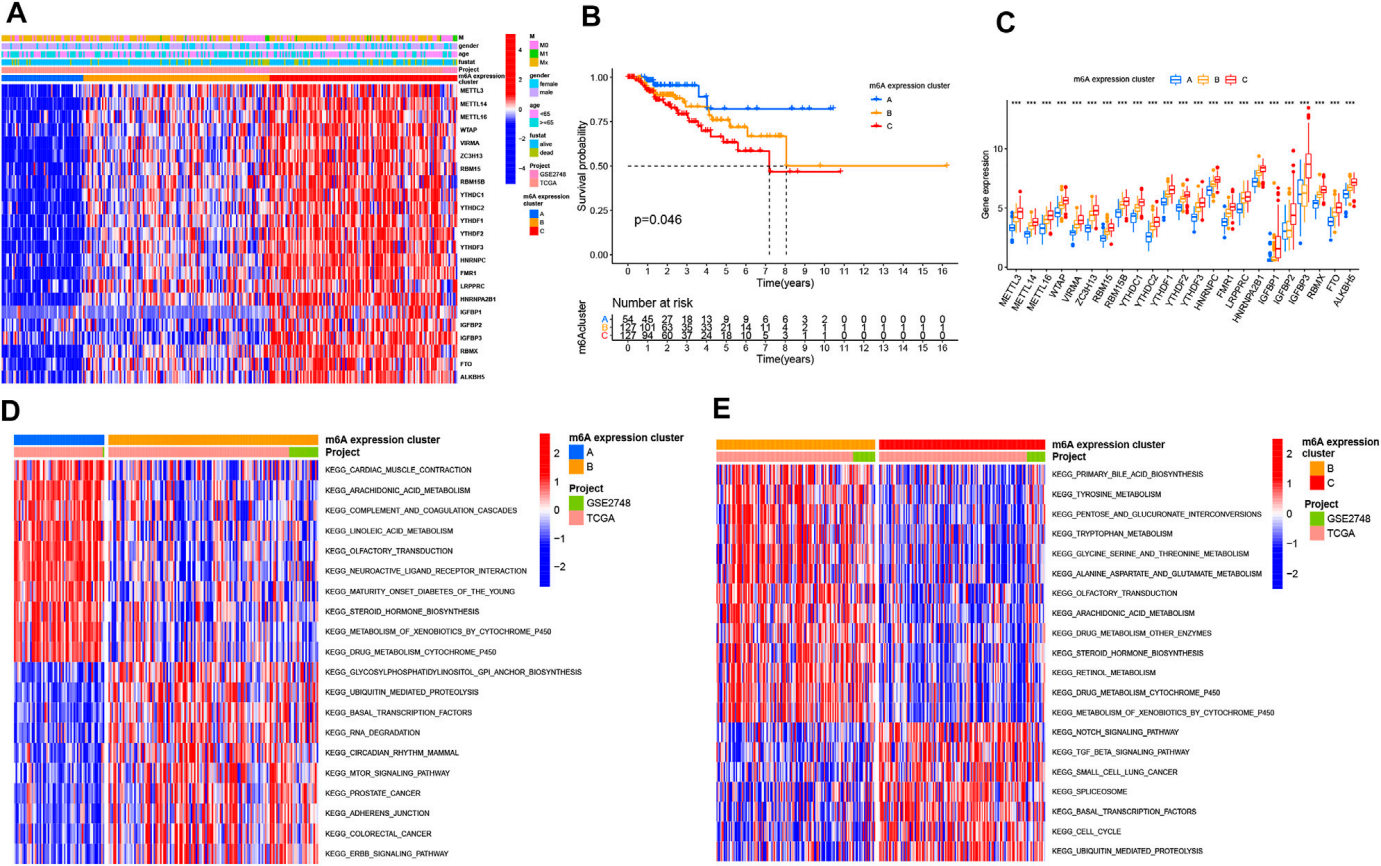
Distinct m^6^A modification patterns and biological features of each cluster. **(A)** Unsupervised clustering of 23 m^6^A genes in TCGA PRCC and GSE2748 data sets. The m^6^A expression cluster, tumor M stage, survival status, gender, and age were used as annotations. Red means high expression of m^6^A genes, and blue represents low expression. The M means metastasis. **(B)** Kaplan–Meier survival analysis showing the OS for the three m^6^A expression clusters based on TCGA PRCC and GSE2748 data sets. A log-rank test was performed. **(C)** Expression pattern of 23 m^6^A genes among three m^6^A expression clusters. The upper and lower ends of the boxes mean interquartile range (IQR) of the values. The lines in the boxes represent each the median value. The asterisks represent the *p* value. (*p < .05; **p < .01; ***p < .001) **(D**,**E)** GSVA enrichment analysis depicting the activation states of the biological processes in three m^6^A expression clusters. In the heat map, red means activated pathways, and blue means inhibited pathways. **(D)** m^6^A expression clusters B vs. A **(E)** m^6^A expression clusters B vs. C.

Based on the expression levels of the 23 m^6^A genes, we classified the PRCC patients by carrying out unsupervised clustering analysis (Supplementary Figures S2A–E). We finally identified three patterns, termed as m^6^A expression clusters A, B and C, which included 56 cases in m^6^A expression cluster A, 128 cases in m^6^A expression cluster B, and 127 cases in m^6^A expression cluster C (Figure 2A). Then, we determined the prognostic values of the three m^6^A modification patterns. According to this analysis, m^6^A expression cluster A showed the most favorable survival (Figure 2B). After combing the TCGA and GEO data sets for comprehensive clinical data from PRCC patients, we made a heat map to visualize the correlation between the three m^6^A expression clusters and clinical characteristics. As shown in the Figure 2A, m^6^A expression cluster C was associated with poor prognosis and enriched in metastatic tumors as well as being associated with patient old age. By comparison, m^6^A expression clusters A and B showed relatively better prognose. We also noted that 23 m^6^A-related genes had relatively high expression levels in m^6^A expression cluster C, followed by m^6^A expression clusters B and A (Figure 2C).

### Biological and TME Cell Infiltration Characteristics in Three m^6^A Modification Patterns

**FIGURE 3:**
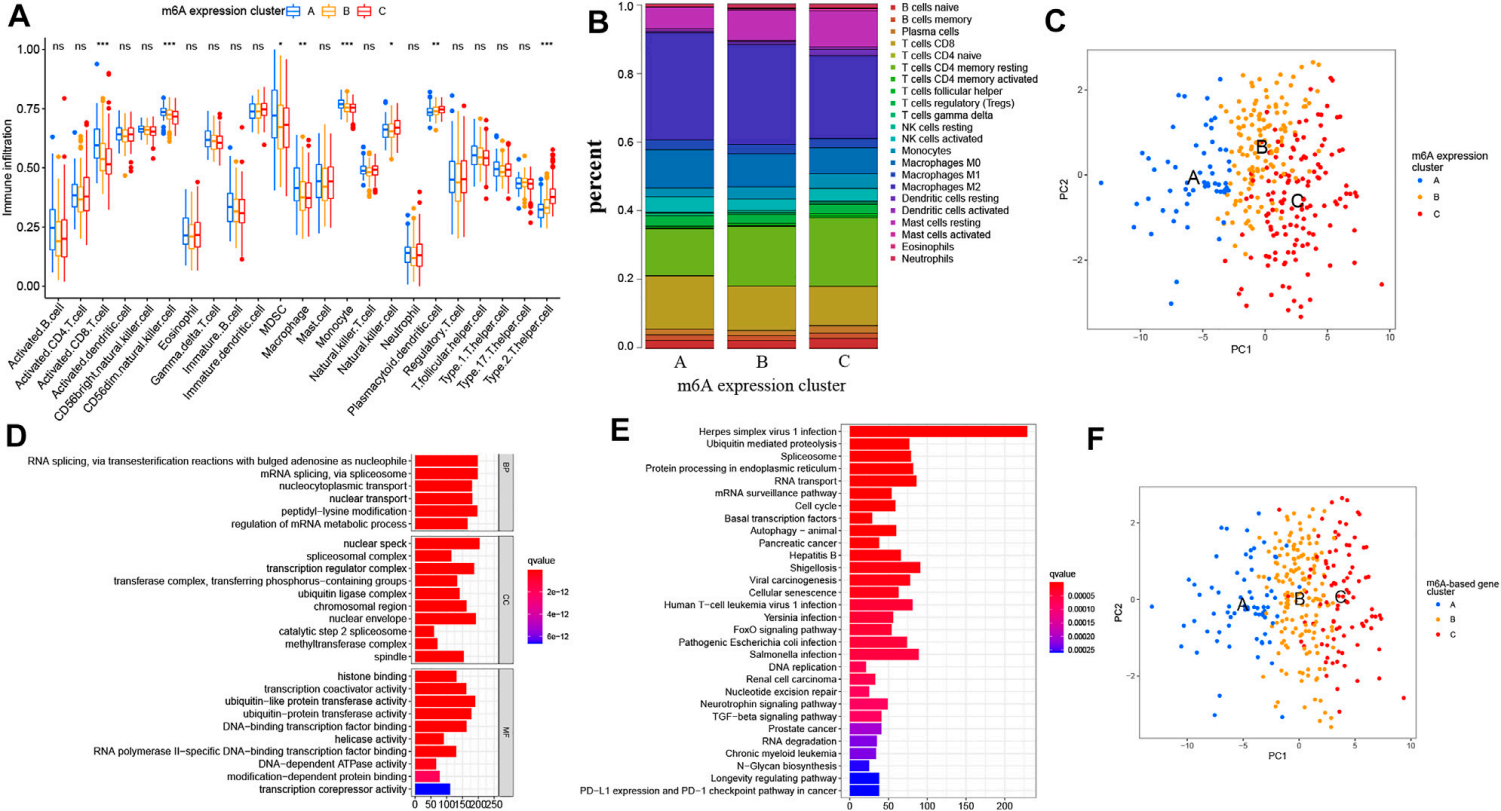
Characteristics of TME cell infiltration in diverse m^6^A expression clusters and the construction of m^6^A gene signatures. **(A)** Abundance of TME-related immune cells in three m^6^A patterns. The histogram shows the expression difference of 23 kinds of immune cells between m^6^A expression cluster A, B, and C. The upper and lower ends of the boxes mean interquartile range (IQR) of the values. The lines in the boxes represent each the median value. The asterisks represent the *p* value. (*p < .05; **p < .01; ***p < .001) **(B)** Proportion of different TME-related immune cells in the three m^6^A patterns as analyzed by CIBERSORT. **(C)** Principal component analysis of PRCC patients in three m^6^A expression clusters, which indicates a remarkable difference between the different modification patterns. **(D**,**E)** Functional analysis for m^6^A-related genes. **(D)** GO enrichment analysis. **(E)** KEGG enrichment analysis. MF means molecular function, CC means cellular component, and BP means biological process. **(F)** Principal component analysis of PRCC patients for the three m^6^A-based gene expression clusters. The left part of each figure indicates the biological functions and signaling pathways. The degree of enrichment is represented by the color depth of each bar plot.

To investigate the biological processes associated with the three types of m^6^A modification patterns, we performed a GSVA analysis. The m^6^A expression cluster A was found to be associated with immune activation processes, such as complement and coagulation cascades. The m^6^A expression cluster B was found to be associated with oncogenic and stromal signaling pathways, including mTOR signaling pathways, ERBB signaling pathways, and adherens junction. The m^6^A expression cluster C was also found to be related with immune-related pathways, such as the Notch signaling pathway (Figures 2D,E). Then, we explored the TME cell infiltration for the different m^6^A expression clusters. The ssGSEA analysis presented that activated CD8^+^ T cells, myeloid-derived suppressor cells, and several innate immune cells, such as macrophages and monocytes, were enriched in m^6^A expression cluster A (Figure 3A). Moreover, m^6^A expression cluster C was associated with natural killer cells, plasmacytoid dendritic cells, and type 2 T helper cells. Afterward, we determined the proportion of immune cells in the three m^6^A expression clusters by using the CIBERSORT algorithm (Figure 3B). However, a significant difference between the different immune cells was not observed. Finally, we used principal component analysis (PCA), which verified significant differences between the three distinct clusters of PRCC patients (Figure 3C).

### Model and Biological Characteristics of the m^6^A Regulators

To further describe the features of the three m^6^A expression clusters, we identified 4780 intersected m^6^A DEGs among the three clusters (Supplementary Figure S2E). Afterward, we analyzed these phenotype-related genes by carrying out KEGG and GO enrichment analyses. The GO analysis revealed a significant enrichment (FDR <0.01) of the methyltransferase complex, RNA methyltransferase activity, and activation of innate immune response (Figure 3D and Supplementary Table S1). The KEGG pathway analysis also indicated that RCC, PD-L1 expression, and the PD-1 checkpoint pathway in cancer were enriched in these selected m^6^A DEGs (Figure 3E and Supplementary Table S2). The above analysis further confirmed the pivotal role played by m^6^A modification in immune regulation as well as RCC. Next, univariate Cox regression analysis was carried out to determine the prognosis-related m^6^A genes. Here, 1285 prognosis-related m^6^A regulators were extracted for unsupervised clustering analysis. With the optimal *k* = 3, three genomic clusters were constructed and named m^6^A-based gene expression clusters A–C (Supplementary Figures S3A–E). A PCA analysis found difference between these three m^6^A-based gene expression clusters as well (Figure 3F). Once again, these results confirmed that diverse m^6^A modification patterns occurred for PRCC.

**FIGURE 4:**
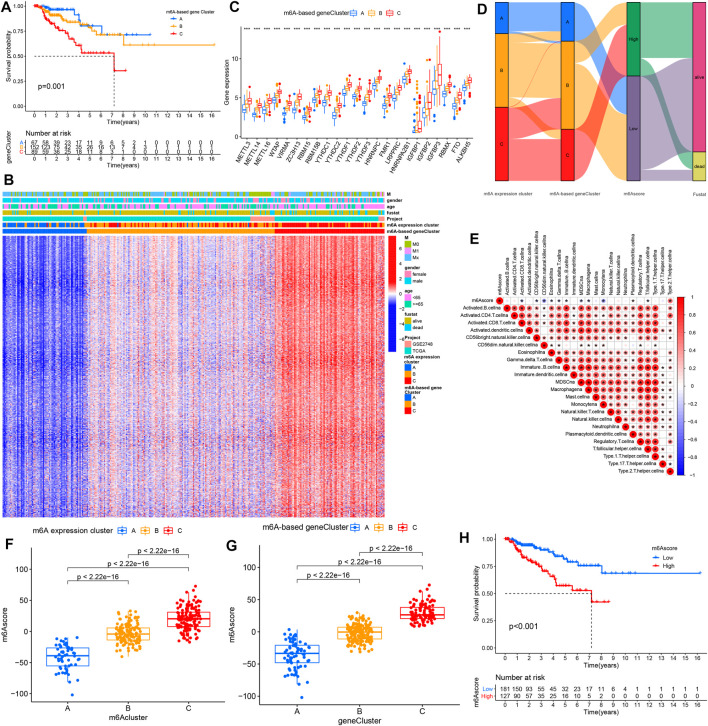
Characteristics of diverse m^6^A-based gene expression clusters. **(A)** Kaplan–Meier survival analysis showed the OS for the three m^6^A-based gene expression clusters based on TCGA PRCC and GSE2748 data set with PRCC. **(B)** Unsupervised clustering of the intersected m^6^A phenotype-related genes in PRCC, which classifies patients into several clusters, termed m^6^A-based gene expression clusters. The m^6^A cluster, tumor M stage, survival status, gender, and age are used as annotations. Red means high expression of m^6^A genes, and blue represents low expression. The M means metastasis. **(C)** Expression pattern of 23 m^6^A genes for the three m^6^A-based gene expression clusters. The histogram indicates the expression level of 23 m^6^A genes between m^6^A-based gene cluster A, B, and C. The upper and lower ends of the boxes mean each the interquartile range (IQR) of values. The lines in the boxes represent median value. The asterisks represented the *p* value. (*p < .05; **p < .01; ***p < .001). **(D)** Alluvial diagram displaying the differences in m^6^A expression clusters, m^6^A-based gene expression clusters, and m^6^Ascore. **(E)** Spearman analyses of the correlations between m^6^Ascore and biological characteristics in the PRCC cohort. **(F**,**G)** Differential analysis of m^6^A score values among **(F)** m^6^A expression clusters and **(G)** m^6^A-based gene expression clusters in TCGA PRCC and GSE2748 data sets. **(H)** K–M analyses for the OS of PRCC patients in high and low m^6^A score groups.

To determine the clinical relevance of these clusters, we evaluated the healthy status among the three m^6^A-based gene expression clusters. The m^6^A-based gene cluster C showed a worse prognosis than did m^6^A-based gene expression clusters A and B (Figure 4A). As shown in Figure 4B, m^6^A-based gene expression cluster C was mainly enriched in metastatic tumors. However, the other clusters were related with alive status as well as nonmetastatic tumor (Figure 4B). The results of the differential analysis of the three clusters validated the pattern of m^6^Agene signatures as well (Figure 4C).

### Evaluation of the m^6^A Modification Patterns Among the m^6^A Regulator Signatures

We employed m^6^A score (a scoring methodology) to quantify and evaluate m^6^A modification patterns. Alterations of each of the PRCC patient’s attributes were visualized by producing and inspecting an alluvial diagram. The results suggest that most of the PRCC samples showing the m6A-based gene expression cluster C were marked with a high m^6^A score and showed poor patient survival (Figure 4D). Then, we assessed the correlations between m^6^A score and biological processes. The m^6^A score was only positively associated with processes involving type 2 T helper cells but negatively correlated with processes involving other immune cells (Figure 4E). Significant differences in the m^6^A score were observed between the three m^6^A-based gene expression clusters as well as between the m^6^A expression clusters. Both of these results presented that m6A expression cluster C and m6A-based gene expression cluster C have the highest m^6^Ascore (Figures 4F,G). Afterward, PRCC patients were divided into two distinct groups with an optimal cutoff value. As shown in Figure 4H, patients with low m^6^A scores showed relatively good survival compared with the high m^6^A score group.

### The m^6^A Modification Model in the Role of Tumor Somatic Mutation and Immunotherapy

**FIGURE 5:**
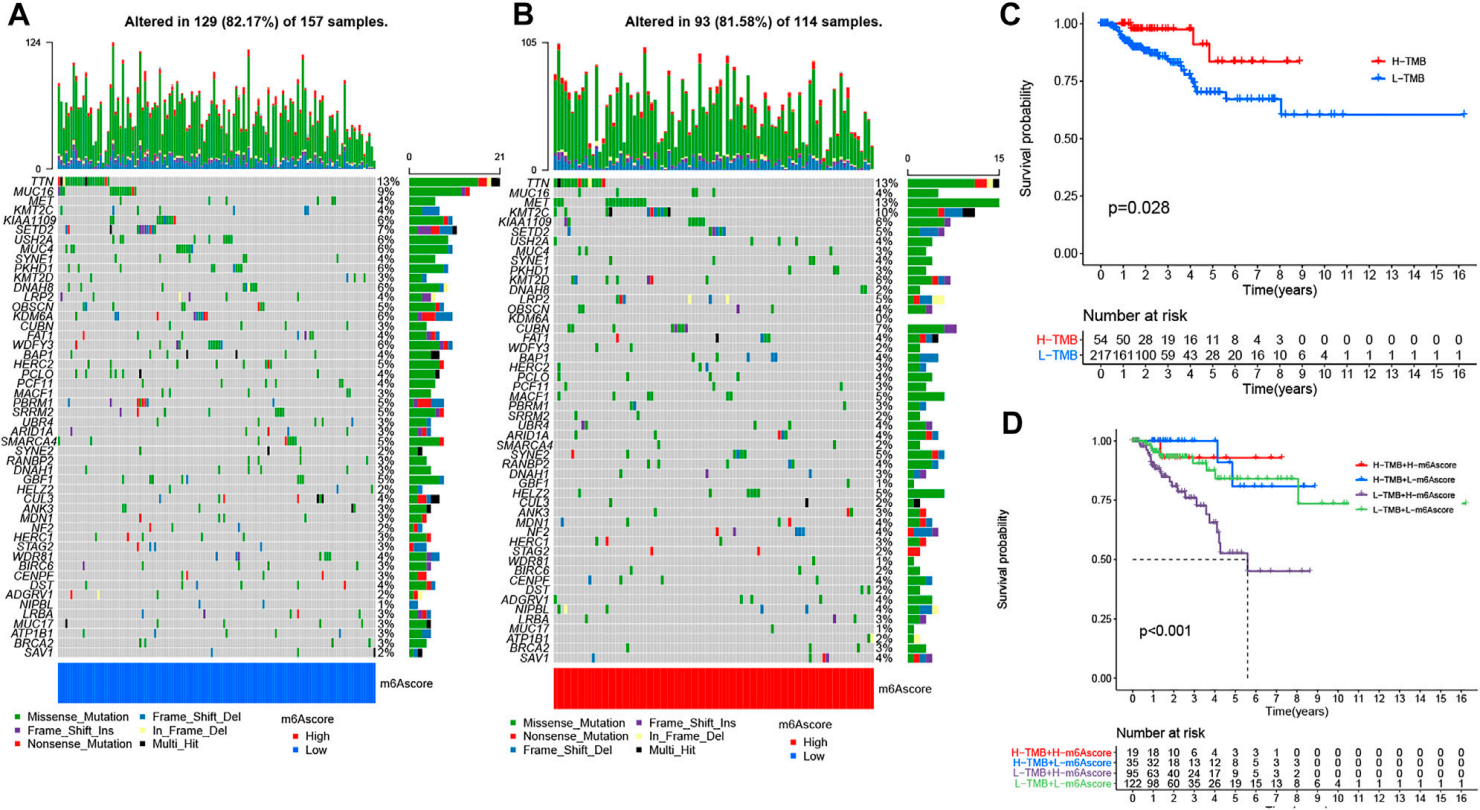
The changes of somatic mutations among distinct m^6^A score groups. **(A**,**B)** Waterfall plot showing The changes of somatic mutations in **(A)** the low-m6A score set and **(B)** high-m6A score group. The bar plot at the upper part of the figure shows TMB. The numbers on the right part of the figure represent the mutation frequency. **(C)** Survival analysis for the OS of patients in high- and low-TMB groups. **(D)** K-M analysis for PRCC patients stratified by both m^6^A score and TMB. MSI-H, high microsatellite instability; MSI-L, low microsatellite instability.

We also analyzed and visualized the somatic mutation profiles of PRCC patients of the high and low m^6^A score groups by using the “maftools” package. Compared with the high m^6^A score set, the low m^6^A score group showed a higher percentage of somatic mutations (Figures 5A,B). A previous study shows an association of high TMB with better survival for most cancers (Xie et al., 2020). Still, a high TMB could improve the prognosis for patients treated with ICIs (Samstein et al., 2019). Considering the significant role of TMB, we tested its prognosis value for PRCC. As observed in the survival plot, the high-TMB set presented improved survival (Figure 5C). Moreover, we found the worst survival for the PRCC patients with both a low-TMB and high m^6^A score (Figure 5D). The above outcome implies that TMB as well as m^6^A score could potentially be used as predictive biomarkers.

**FIGURE 6:**
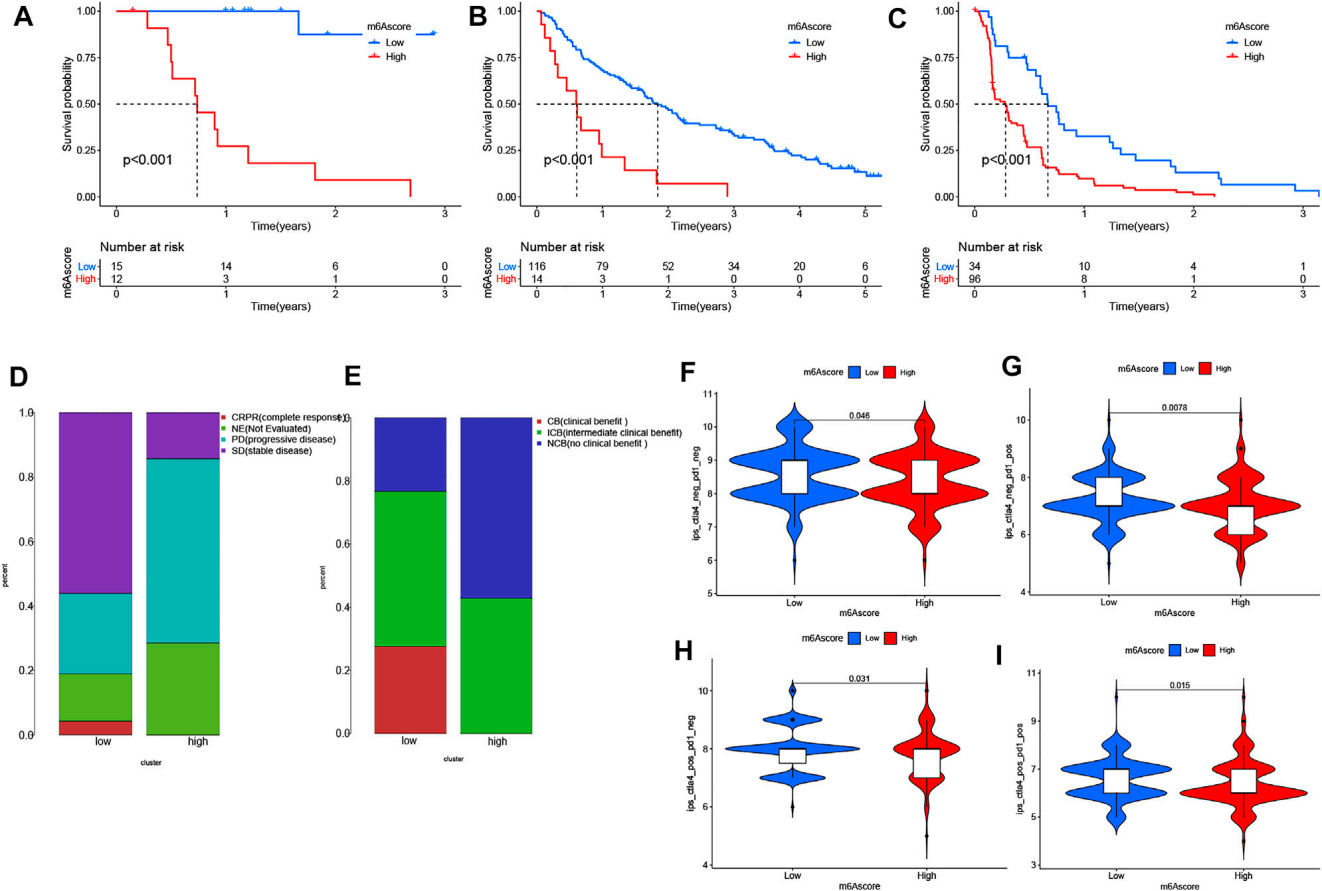
The role of distinct m^6^A modification patterns in immunotherapy. **(A–** **C)** Results showing the associations of m6A score was negatively associated with OS and PFS following **(A**,**B)** anti-PD-1 therapy or **(C)** use of mTOR inhibitors. Negative associations were observed in both cases. **(D**,**E)** Proportions of PRCC patients with **(D)** an immunotherapy response and **(E)** clinical benefit in the two m6Ascore sets. **(F–** **I)** Relationship between m6A score and immunotherapeutic response under PD-1 and CTLA-4 expressions: **(F)** negative PD-1 and CTLA-4. **(G)** positive PD-1 and negative CTLA-4. **(H)** positive CTLA-4 and negative PD-1. **(I)** positive PD-1 and CTLA-4.

Next, we interrogated the clinical value of the m^6^A modification model in immunotherapy (including PD-1 blockade and mTOR inhibitor). In the PD-1 blockade cohort (GSE78220), patients with low m^6^A scores showed improved overall survival (OS) (Figure 6A). In addition, in the anti-mTOR group, there was a significant difference in OS as well as progression free survival (PFS) between low and high m^6^A score groups. The therapeutic advantages of the mTOR inhibitor was observed in the low m^6^A score group (Figures 6B,C). Moreover, in light of unsatisfactory outcomes from tumor therapy, we queried whether m^6^A score could affect the therapeutic efficacy. The poor outcome of overall response rate and clinical benefit was correlated with high m^6^A score (Figures 6D,E). Finally, we used the m^6^A score to predict the reaction to immunotherapy efficacy. After downloading the immunotherapy fraction data from the Cancer Immunome Database (TCIA), we compared the predictive abilities of the m^6^A scores of the two m^6^A score groups. Patients with low m^6^A score values showed significantly better reactions to anti-CTLA-4 and anti-PD-1 therapy (Figures 6F–I).

### Biological Validation of Significant m^6^A Regulators and Immune Cell Markers

**FIGURE 7:**
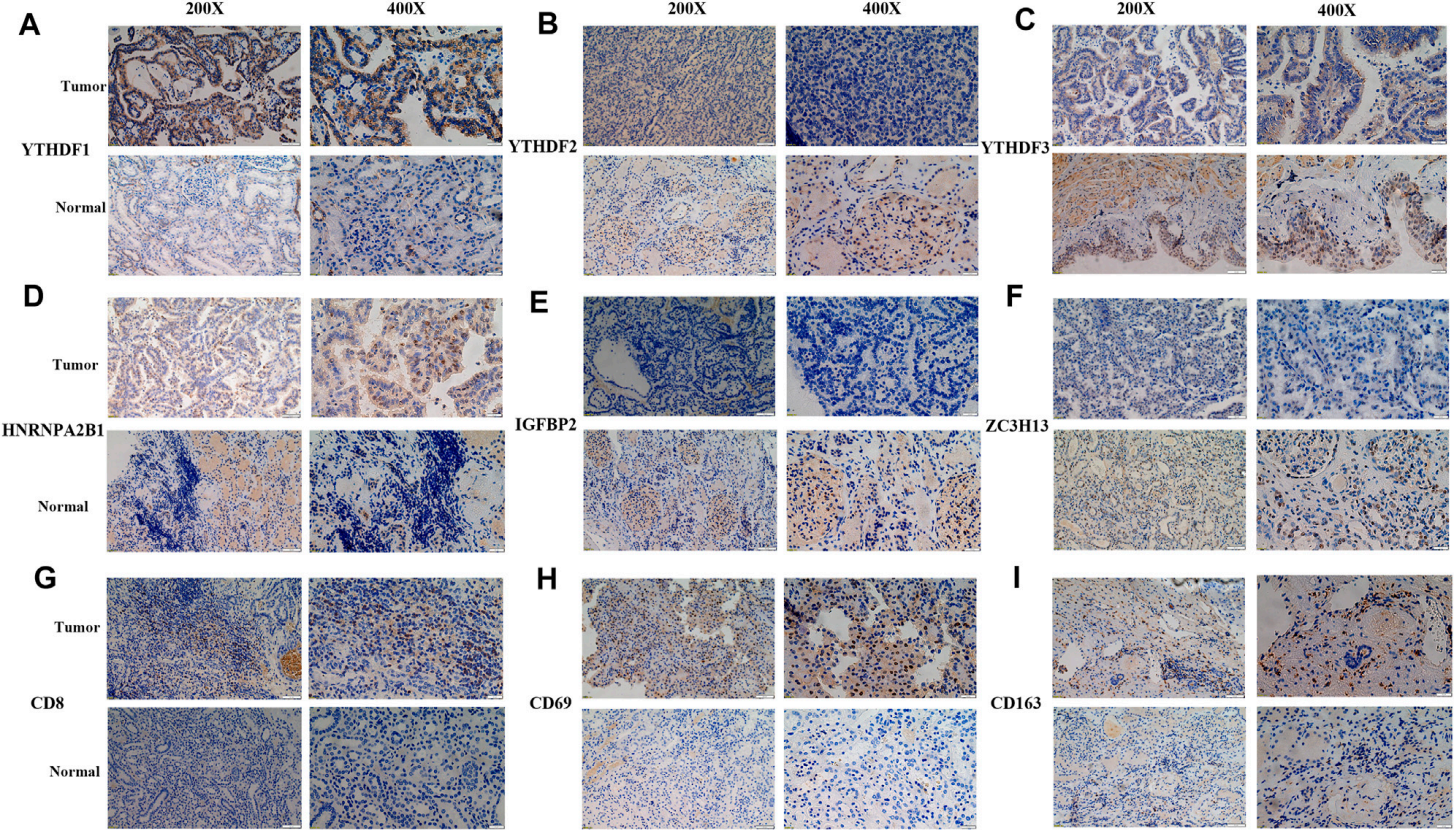
Representative IHC images of significant m^6^A regulators and immune cell markers in PRCC samples. **(A–F)** IHC patterns for selected m^6^A regulators in normal and tumor samples. **(G–I)** IHC patterns for three immune cell markers in normal and tumor samples. Bar, 50 and 200 μm.

The robustness of m^6^A regulators as biomarkers was verified using primary PRCC clinical samples from the Shandong Provincial Hospital affiliated with Shandong First Medical University. We selected six m^6^A genes from the DEGs and five immune cell markers for the following validation. The IHC images acquired of immune cell markers showed weak staining for CD8, CD69, and CD163 in normal renal tissue (Figures 7G–I). Tumor tissue staining of YTHDF1 and HNRNPA2B1 showed moderate staining in the nucleus, and negative staining was observed in the normal tissues (Figures 7A,D). In normal kidney samples, moderate staining for ZC3H13 and YTHDF2 were observed in the nucleus. Regarding the YTHDF3 and IGFBP2, strong staining was positive on the cytoplasm (Figures 7B,C,E,F). However, weak staining patterns for ZC3H13, YTHDF2, YTHDF3, and IGFBP2 were observed in PRCC tissues (Figures 7B,C,E,F). These unique IHC staining patterns further confirmed the above results and illustrated that these selected m^6^A regulators could be used to predict clinical outcomes.

## Discussion

The m^6^A modification plays a pivotal role in tumorigenesis, tumor development, progression, and prognosis (He et al., 2019). Previous studies show the m^6^A modification displaying dual suppressive and promotive functions in various tumors (He et al., 2018; Wang et al., 2018). However, there are few studies of the m^6^A modification for RCC (especially PRCC) initiation, progression, and therapy. The TME is a potential regulator of cancer progression and a source of therapeutic targets. In the complex TME, immune and stromal cells play significant roles in cancer development (Quail and Joyce, 2013; Ho et al., 2020). Currently, knowledge of the kidney TME is restricted to only a few different tumor types and lacks comprehensive analysis. Therefore, in this study, we focused our attention on the role of m^6^A modification in the TME of PRCC and aimed to unravel the potential functions of this modification and contribute to obtaining a deeper understanding of antitumor immune effects of the TME in PRCC.

CNV is one of the most important somatic aberrations in cancer, and several studies find significant associations between CNVs and cancers (Speleman et al., 2008; Shlien and Malkin, 2009; Beroukhim et al., 2010). Based on 23 m^6^A genes and PRCC copy-number profiles, we explored the alteration of m^6^A genes in PRCC. The mutations of the m^6^A regulators occurred relatively infrequently in PRCC, but CNV deletion was a common event. Then, on the basis of clustering analysis, we identified three different m^6^A expression clusters in PRCC. In 2017, Chen DS et al. proposed three types of cancer-immune phenotypes, namely, immune-inflamed, immune-excluded, and immune-desert phenotypes (Speleman et al., 2008; Shlien and Malkin, 2009; Beroukhim et al., 2010). The immune-inflamed phenotype is characterized by the presence of CD4^+^ T, CD8^+^ T, myeloid, and monocytc cells in the TME, which is positioned near the tumor cells (Herbst et al., 2014; Turley et al., 2015). The immune-excluded phenotype also involves the presence of many immune cells, but with these cell, located mainly surrounding the stroma instead of the nest of the tumor (Joyce and Fearon, 2015; Hegde et al., 2016). The immune-desert phenotype presents a paucity of CD8^+^ T cells in both tumor parenchyma and stroma with this paucity being a feature of a noninflamed TME (Gajewski et al., 2013; Kim and Chen, 2016). In our current study, we found an enrichment of activated CD8^+^ T cells, myeloid-derived suppressor cells, macrophages, and monocytes in m^6^A expression cluster A, an association of the m^6^A expression cluster B with adherens junction, and m^6^A expression cluster C showing the presence of natural killer and plasmacytoid dendritic cells. Due to the presence of CD8 expressing T cells and other myeloid cells as well as monocytes, the m^6^A expression cluster A showed improved survival.

Then, we identified the intersected DEGs between diverse m^6^A expression clusters and assessed the potential biological functions of these genes and the pathways used by them. Our results show a significant enrichment of these DEGs in m^6^A-, immune- and immunotherapy-related biological functions and pathways. Moreover, we chose T cell (CD8, CD69) and macrophage markers (CD163) as well as differentially expressed m^6^A regulators to validate the clinical application using primary PRCC samples from our hospital, and the results further confirm the prognostic value in clinical application. To limit the individual heterogeneity, we utilized m^6^A score to quantify and evaluate m^6^A modification patterns. Similar to the results of previous research, the m^6^A expression cluster C and m^6^A expression cluster A in the current work presented, respectively, the highest and lowest m^6^A score in PRCC. The K-M survival curve illustrates a better OS and better prognosis associated with m^6^A-based gene expression cluster A than with m^6^A-based gene expression cluster C. These results suggest that the m^6^A scoring system could be applied to determine distinct immune phenotypes and m^6^A modification patterns.

Somatic mutation was detected between high- and low-m^6^A score groups as well. The low m^6^A score group had a high TMB with high TMB associated with better survival for PRCC patients. A similar trend was found in studies involving melanoma and osteosarcoma (Aoude et al., 2020; Xie et al., 2020). Still, a high TMB appears to indicate a better prognosis for patients receiving ICIs for treating various types of tumors (Snyder et al., 2014; Rizvi et al., 2015; Van Allen et al., 2015; Rosenberg et al., 2016). These findings suggest better immunotherapeutic outcomes for the low m^6^A score group than for the high m^6^A score group. In light of the disappointing outcomes from immunotherapy (including anti-PD-1 therapy and mTOR inhibitors) to date (Larkin et al., 2015; Postow et al., 2015; Rotte et al., 2018; de Vries-Brilland et al., 2020), we sought to determine whether m^6^A score could serve as a biomarker to stratify patients with different levels of immune-responsiveness to tumors. By utilizing GSE78220 (PD-1 blockade cohort) and the anti-mTOR group (Hugo et al., 2016; Braun et al., 2020), we showed an association between a low m^6^A score and improve OS and PFS time. Thus, distinct m^6^A modification patterns may impact the efficacy of immunotherapy, and m^6^A score has potential clinical value in evaluating the efficacy of therapeutic.

To improve the outcome for PRCC patients, access to accurate and efficient biomarkers is indispensable. Therefore, we investigated the TME and m^6^A-related genes to reveal the associated immune cells and molecular mechanism as well as clinical value. This investigation suggests that diverse m^6^A modification patterns could affect the complexity of the PRCC TME. Moreover, the established m^6^A score was indicated by our results to have great potential as a predictive indicator to assess the distinct m^6^A modification patterns and prognose of PRCC patients. More importantly, given the high variety of responses to immunotherapy, the m^6^A score may be utilized to evaluate how tumors might react to being exposed to an immunotherapy (including anti-PD-1 therapy and mTOR inhibitors). We do note that the relatively small number of PRCC patients receiving immunotherapy may affect the predictive ability of m^6^A score. Therefore, in future investigations, expression data and clinical information from our medical center will be collected. Further experiments *in vivo* and *in vitro* will also be implemented to confirm the molecular mechanism of m^6^A-related regulators in the PRCC TME. Nevertheless, the study we carried out has enhanced our understanding of TME characteristics and improved the therapeutic landscape for PRCC patients.

## Data Availability

The original contributions presented in the study are included in the article/Supplementary Material, further inquiries can be directed to the corresponding authors.

## Abbreviations

ccRCC, clear cell renal cell carcinoma; CNV, Copy number variation; DEGs, Differentially expressed genes; GEO, Gene-Expression Omnibus; GSVA, Gene set variation analysis; ICI, Immunological checkpoint inhibitor; m^6^A, N6-methyladenosine; PCA, Principal component analysis; PRCC, papillary renal cell carcinoma; ssGSEA, Single-sample gene-set enrichment analysis; TCGA, The Cancer Genome Atlas; TCIA, The Cancer Immunome Atlas; TMB, Tumor mutation burden; TME, tumor microenvironment.
