# Supplementary figures and images for: Identification of New m6A Methylation Modification Patterns and Tumor Microenvironment Infiltration Landscape that Predict Clinical Outcomes for Papillary Renal Cell Carcinoma Patients
Source: Front Cell Dev Biol. 2022 Mar 17;10:818194. doi: 10.3389/fcell.2022.818194 (PMC8968637; doi:10.3389/fcell.2022.818194)

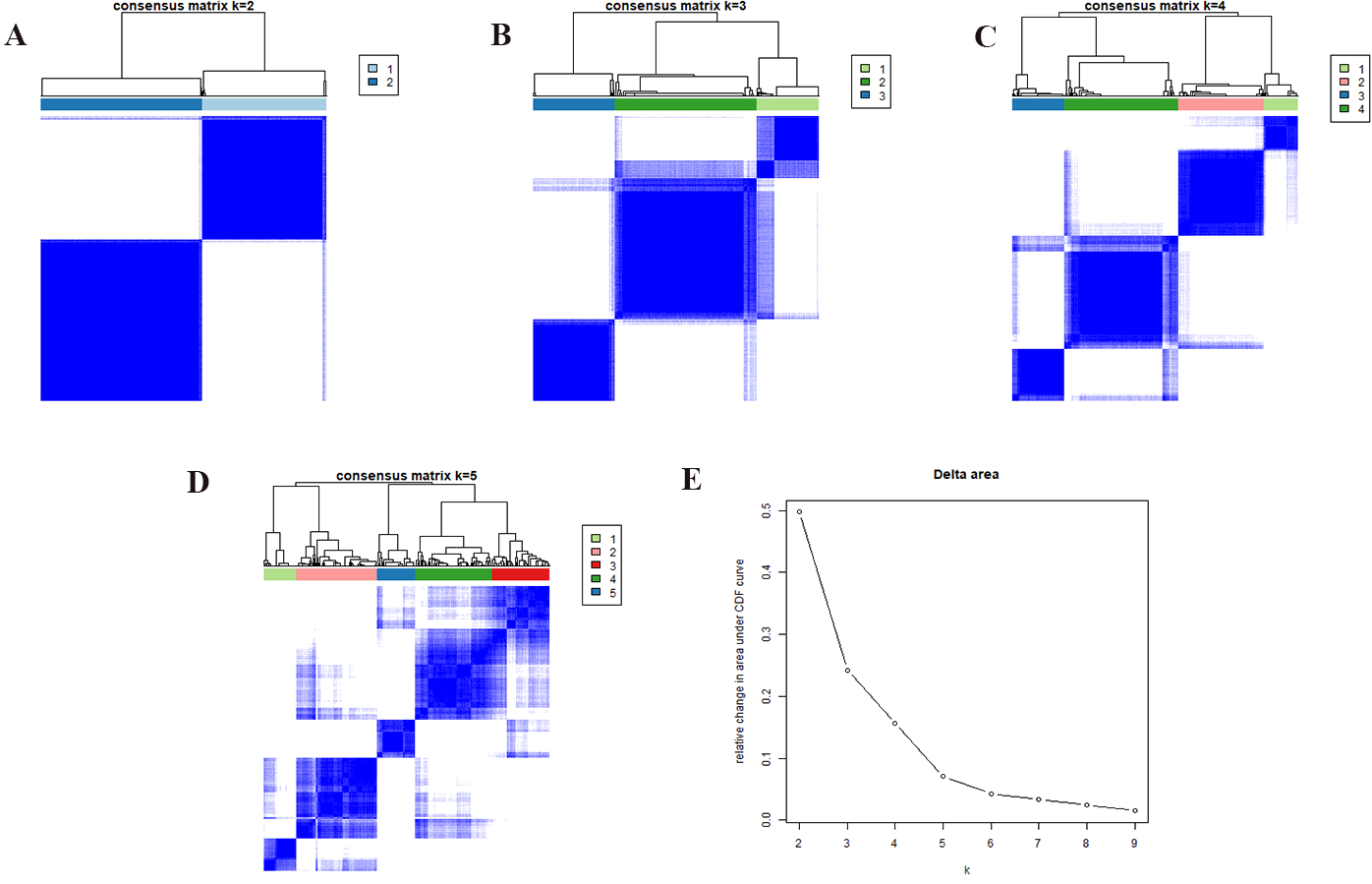

Supplement: Supplementary file 1 [file Image3.TIF]

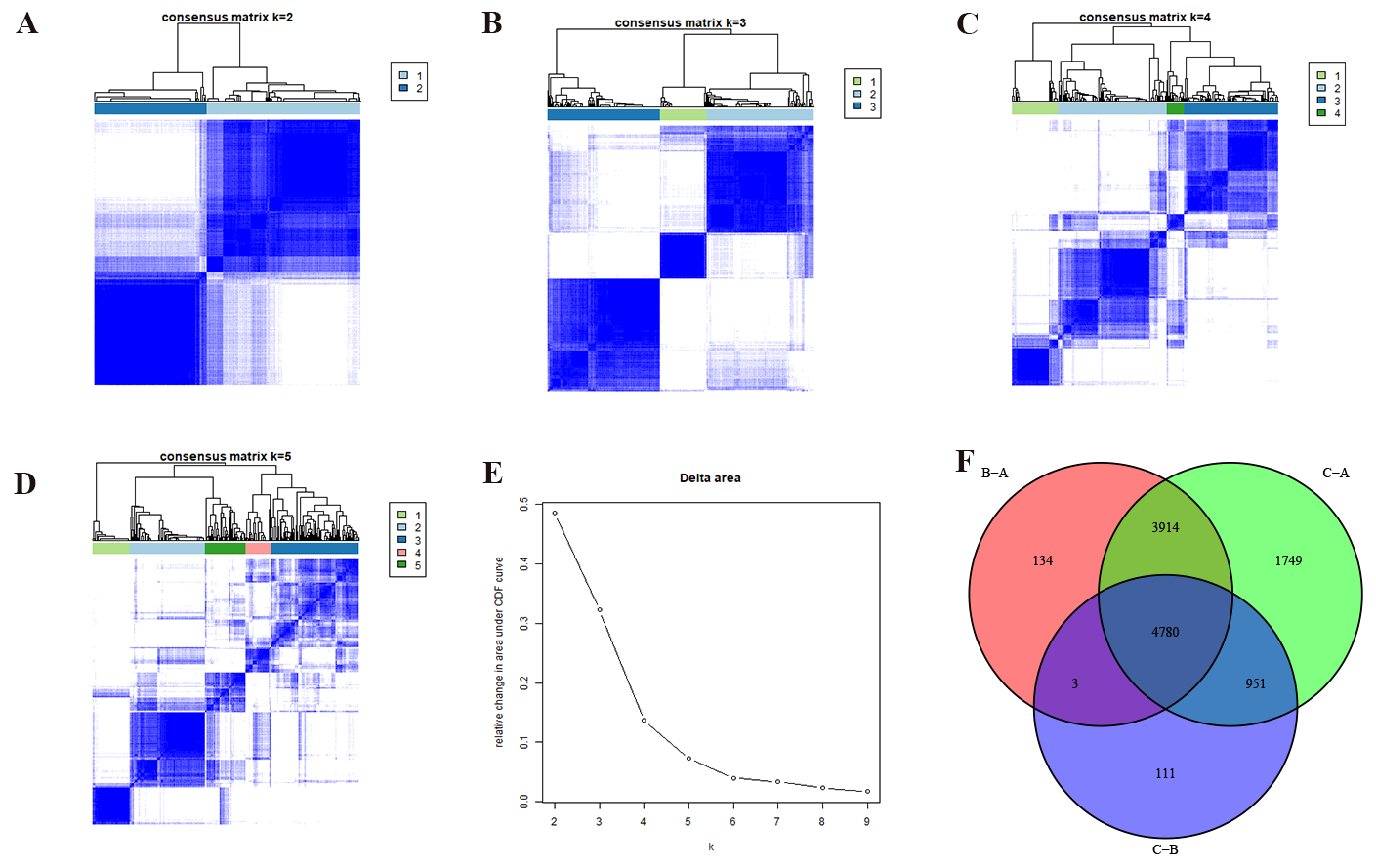

Supplement: Supplementary file 2 [file Image2.TIF]

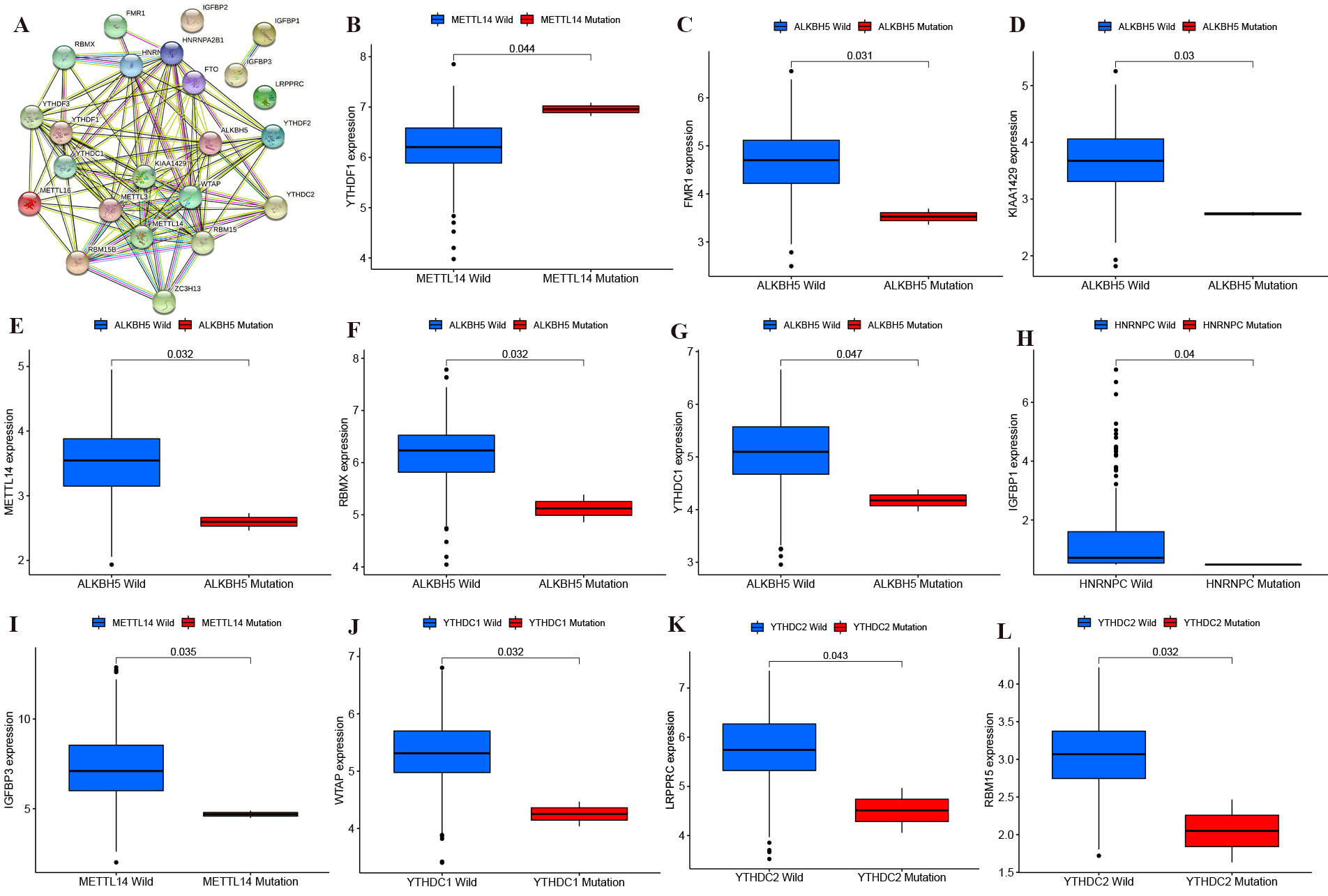

Supplement: Supplementary file 3 [file Image1.TIF]
