# Supplementary material for: Identification of New m6A Methylation Modification Patterns and Tumor Microenvironment Infiltration Landscape that Predict Clinical Outcomes for Papillary Renal Cell Carcinoma Patients
Source: Front Cell Dev Biol. 2022 Mar 17;10:818194. doi: 10.3389/fcell.2022.818194 (PMC8968637; doi:10.3389/fcell.2022.818194)
Supplement: Supplementary file 4 [file DataSheet1.PDF]

Supplementary Material

1 Supplementary Figures

Supplementary Figure. 1 Correlation between 24 m^{6}A-related gene expression. A PPI network of 23 m6A-related genes. **B-L** the expression variation of m6A-related genes under mutated and wild genetic alterations.

Supplementary Figure. 2 A-D Consensus matrices of PRCC set for k = 2-5 in different m6Aclusters. **E** Criteria for number of categories. **F** The intersected phenotype-related genes.

Supplementary Figure. 3 A-D Consensus matrices of PRCC set for k = 2-5 in different m6Agene clusters. **E** Criteria for number of categories.

Supplementary Table. 1 The results of significant GO functions and GO terms.

ONTOLOGY	ID	Description	pvalue	p.adjust	qvalue	Count
BP	GO:0016569	covalent chromatin modification	2.43E-47	1.51E-43	1.22E-43	252
BP	GO:0016570	histone modification	2.39E-46	7.45E-43	6.00E-43	243
BP	GO:0008380	RNA splicing	4.14E-43	8.60E-40	6.93E-40	243
BP	GO:0000375	RNA splicing, via transesterification reactions	1.88E-36	2.93E-33	2.36E-33	200
BP	GO:0000377	RNA splicing, via transesterification reactions with bulged adenosine as nucleophile	6.37E-36	6.62E-33	5.33E-33	198
BP	GO:0000398	mRNA splicing, via spliceosome	6.37E-36	6.62E-33	5.33E-33	198
BP	GO:0006913	nucleocytoplasmic transport	4.50E-33	3.97E-30	3.20E-30	180
BP	GO:0051169	nuclear transport	5.10E-33	3.97E-30	3.20E-30	181
BP	GO:0018205	peptidyl-lysine modification	8.95E-32	6.20E-29	4.99E-29	197
BP	GO:1903311	regulation of mRNA metabolic process	5.41E-29	3.37E-26	2.71E-26	166
BP	GO:0006260	DNA replication	8.89E-29	5.04E-26	4.06E-26	147
BP	GO:0006403	RNA localization	2.61E-28	1.35E-25	1.09E-25	130
BP	GO:0051236	establishment of RNA localization	1.85E-26	8.87E-24	7.14E-24	114
BP	GO:0051168	nuclear export	2.58E-26	1.15E-23	9.26E-24	113
BP	GO:0043161	proteasome-mediated ubiquitin-dependent protein catabolic process	5.86E-26	2.43E-23	1.96E-23	193
BP	GO:0050657	nucleic acid transport	6.68E-26	2.45E-23	1.97E-23	112

BP	GO:0050658	RNA transport	6.68E-26	2.45E-23	1.97E-23	112
BP	GO:0010498	proteasomal protein catabolic process	7.26E-26	2.51E-23	2.02E-23	212
BP	GO:0033044	regulation of chromosome organization	1.47E-24	4.83E-22	3.89E-22	164
BP	GO:0050684	regulation of mRNA processing	3.21E-24	9.99E-22	8.05E-22	87
BP	GO:0006405	RNA export from nucleus	2.52E-23	7.48E-21	6.02E-21	85
BP	GO:0006611	protein export from nucleus	7.20E-23	2.04E-20	1.64E-20	102
BP	GO:0015931	nucleobase-containing compound transport	3.49E-22	9.46E-20	7.61E-20	124
BP	GO:0051028	mRNA transport	3.13E-21	8.14E-19	6.55E-19	89
BP	GO:0071426	ribonucleoprotein complex export from nucleus	1.49E-20	3.71E-18	2.99E-18	78
BP	GO:0048193	Golgi vesicle transport	2.04E-20	4.90E-18	3.94E-18	164
BP	GO:0043414	macromolecule methylation	2.95E-20	6.59E-18	5.31E-18	144
BP	GO:0071166	ribonucleoprotein complex localization	2.96E-20	6.59E-18	5.31E-18	78
BP	GO:0043484	regulation of RNA splicing	1.13E-19	2.43E-17	1.96E-17	80
BP	GO:0044839	cell cycle G2/M phase transition	1.30E-19	2.70E-17	2.18E-17	128
BP	GO:1902275	regulation of chromatin organization	1.47E-19	2.95E-17	2.37E-17	99
BP	GO:0031503	protein-containing complex localization	1.55E-19	3.02E-17	2.43E-17	133
BP	GO:0000209	protein polyubiquitination	3.74E-19	7.07E-17	5.69E-17	142
BP	GO:0006261	DNA-dependent DNA replication	7.89E-19	1.45E-16	1.16E-16	85

BP	GO:0072331	signal transduction by p53 class mediator	1.90E-18	3.38E-16	2.73E-16	126
BP	GO:0022613	ribonucleoprotein complex biogenesis	2.15E-18	3.73E-16	3.00E-16	191
BP	GO:0048024	regulation of mRNA splicing, via spliceosome	2.54E-18	4.28E-16	3.45E-16	63
BP	GO:0016573	histone acetylation	2.97E-18	4.87E-16	3.92E-16	86
BP	GO:0006406	mRNA export from nucleus	5.15E-18	8.03E-16	6.47E-16	68
BP	GO:0071427	mRNA-containing ribonucleoprotein complex export from nucleus	5.15E-18	8.03E-16	6.47E-16	68
BP	GO:0000086	G2/M transition of mitotic cell cycle	7.04E-18	1.07E-15	8.62E-16	118
BP	GO:0018394	peptidyl-lysine acetylation	9.92E-18	1.47E-15	1.19E-15	90
BP	GO:0031056	regulation of histone modification	1.37E-17	1.99E-15	1.60E-15	80
BP	GO:0006352	DNA-templated transcription, initiation	1.54E-17	2.18E-15	1.76E-15	118
BP	GO:2001252	positive regulation of chromosome organization	3.06E-17	4.23E-15	3.41E-15	91
BP	GO:0043543	protein acylation	3.24E-17	4.39E-15	3.53E-15	117
BP	GO:0018393	internal peptidyl-lysine acetylation	4.11E-17	5.45E-15	4.39E-15	86
BP	GO:0006473	protein acetylation	7.90E-17	1.03E-14	8.26E-15	101
BP	GO:0070646	protein modification by small protein removal	1.00E-16	1.28E-14	1.03E-14	133
BP	GO:0006367	transcription initiation from RNA polymerase II promoter	1.19E-16	1.48E-14	1.19E-14	95
BP	GO:0034470	ncRNA processing	1.38E-16	1.69E-14	1.36E-14	160
BP	GO:0007098	centrosome cycle	1.68E-16	2.02E-14	1.63E-14	71

BP	GO:0032259	methylation	1.83E-16	2.15E-14	1.73E-14	154
BP	GO:0044786	cell cycle DNA replication	1.90E-16	2.19E-14	1.76E-14	49
BP	GO:1901987	regulation of cell cycle phase transition	2.60E-16	2.95E-14	2.37E-14	189
BP	GO:0000380	alternative mRNA splicing, via spliceosome	2.90E-16	3.23E-14	2.60E-14	51
BP	GO:0031023	microtubule organizing center organization	3.36E-16	3.67E-14	2.96E-14	74
BP	GO:0006475	internal protein amino acid acetylation	4.77E-16	5.13E-14	4.13E-14	86
BP	GO:0006354	DNA-templated transcription, elongation	1.27E-15	1.34E-13	1.08E-13	65
BP	GO:0051170	import into nucleus	1.57E-15	1.63E-13	1.31E-13	84
BP	GO:0097711	ciliary basal body-plasma membrane docking	1.78E-15	1.82E-13	1.46E-13	58
BP	GO:0016571	histone methylation	2.02E-15	2.04E-13	1.64E-13	75
BP	GO:0051656	establishment of organelle localization	2.54E-15	2.51E-13	2.02E-13	162
BP	GO:1901990	regulation of mitotic cell cycle phase transition	3.03E-15	2.95E-13	2.38E-13	175
BP	GO:0006479	protein methylation	3.85E-15	3.64E-13	2.93E-13	89
BP	GO:0008213	protein alkylation	3.85E-15	3.64E-13	2.93E-13	89
BP	GO:0034968	histone lysine methylation	4.20E-15	3.90E-13	3.14E-13	65
BP	GO:0031570	DNA integrity checkpoint	4.59E-15	4.21E-13	3.39E-13	81
BP	GO:0006338	chromatin remodeling	4.77E-15	4.31E-13	3.47E-13	103
BP	GO:0034504	protein localization to nucleus	4.98E-15	4.43E-13	3.57E-13	117

BP	GO:0140056	organelle localization by membrane tethering	6.76E-15	5.94E-13	4.78E-13	85
BP	GO:0000077	DNA damage checkpoint	1.06E-14	9.15E-13	7.37E-13	76
BP	GO:0031124	mRNA 3'-end processing	1.28E-14	1.10E-12	8.82E-13	58
BP	GO:1901796	regulation of signal transduction by p53 class mediator	1.97E-14	1.64E-12	1.32E-12	88
BP	GO:0000381	regulation of alternative mRNA splicing, via spliceosome	1.97E-14	1.64E-12	1.32E-12	44
BP	GO:1903320	regulation of protein modification by small protein conjugation or removal	3.00E-14	2.43E-12	1.96E-12	105
BP	GO:0016925	protein sumoylation	3.04E-14	2.43E-12	1.96E-12	51
BP	GO:0031331	positive regulation of cellular catabolic process	3.04E-14	2.43E-12	1.96E-12	147
BP	GO:0009314	response to radiation	3.90E-14	3.08E-12	2.48E-12	173
BP	GO:0010256	endomembrane system organization	4.20E-14	3.27E-12	2.64E-12	172
BP	GO:0071103	DNA conformation change	5.17E-14	3.98E-12	3.20E-12	147
BP	GO:0006606	protein import into nucleus	5.64E-14	4.29E-12	3.45E-12	74
BP	GO:1902749	regulation of cell cycle G2/M phase transition	8.49E-14	6.35E-12	5.11E-12	98
BP	GO:0000075	cell cycle checkpoint	8.55E-14	6.35E-12	5.11E-12	99
BP	GO:0022406	membrane docking	9.15E-14	6.71E-12	5.40E-12	86
BP	GO:0033260	nuclear DNA replication	9.33E-14	6.77E-12	5.45E-12	41
BP	GO:0006368	transcription elongation from RNA polymerase II promoter	1.21E-13	8.70E-12	7.00E-12	51
BP	GO:0009896	positive regulation of catabolic process	1.94E-13	1.38E-11	1.11E-11	164

BP	GO:0016579	protein deubiquitination	2.05E-13	1.44E-11	1.16E-11	120
BP	GO:0006997	nucleus organization	2.99E-13	2.07E-11	1.67E-11	68
BP	GO:0010212	response to ionizing radiation	3.52E-13	2.41E-11	1.94E-11	74
BP	GO:0044843	cell cycle G1/S phase transition	4.12E-13	2.79E-11	2.25E-11	124
BP	GO:0098781	ncRNA transcription	5.61E-13	3.76E-11	3.03E-11	59
BP	GO:0016050	vesicle organization	6.15E-13	4.08E-11	3.28E-11	132
BP	GO:0010948	negative regulation of cell cycle process	6.73E-13	4.42E-11	3.56E-11	143
BP	GO:0034660	ncRNA metabolic process	8.13E-13	5.28E-11	4.25E-11	177
BP	GO:0031060	regulation of histone methylation	8.63E-13	5.55E-11	4.47E-11	42
BP	GO:0010389	regulation of G2/M transition of mitotic cell cycle	9.98E-13	6.35E-11	5.11E-11	90
BP	GO:0000082	G1/S transition of mitotic cell cycle	1.01E-12	6.36E-11	5.12E-11	117
BP	GO:0032392	DNA geometric change	1.09E-12	6.80E-11	5.47E-11	63
BP	GO:1905269	positive regulation of chromatin organization	1.13E-12	6.96E-11	5.60E-11	57
BP	GO:0044782	cilium organization	1.50E-12	9.17E-11	7.38E-11	149
BP	GO:0000723	telomere maintenance	1.60E-12	9.63E-11	7.76E-11	78
BP	GO:0007030	Golgi organization	1.61E-12	9.63E-11	7.76E-11	71
BP	GO:1903313	positive regulation of mRNA metabolic process	1.64E-12	9.73E-11	7.83E-11	47
BP	GO:0033157	regulation of intracellular protein transport	2.02E-12	1.19E-10	9.59E-11	107

BP	GO:0042795	snRNA transcription by RNA polymerase II	2.11E-12	1.23E-10	9.90E-11	44
BP	GO:0060271	cilium assembly	2.30E-12	1.33E-10	1.07E-10	143
BP	GO:0075733	intracellular transport of virus	2.48E-12	1.42E-10	1.14E-10	38
BP	GO:1903322	positive regulation of protein modification by small protein conjugation or removal	3.04E-12	1.72E-10	1.39E-10	68
BP	GO:1900034	regulation of cellular response to heat	3.15E-12	1.77E-10	1.42E-10	47
BP	GO:0009301	snRNA transcription	4.22E-12	2.35E-10	1.89E-10	44
BP	GO:0046794	transport of virus	4.27E-12	2.36E-10	1.90E-10	39
BP	GO:0042254	ribosome biogenesis	4.40E-12	2.41E-10	1.94E-10	121
BP	GO:0097064	ncRNA export from nucleus	4.71E-12	2.55E-10	2.06E-10	29
BP	GO:0051031	tRNA transport	4.92E-12	2.60E-10	2.09E-10	28
BP	GO:0006409	tRNA export from nucleus	4.92E-12	2.60E-10	2.09E-10	27
BP	GO:0071431	tRNA-containing ribonucleoprotein complex export from nucleus	4.92E-12	2.60E-10	2.09E-10	27
BP	GO:0001701	in utero embryonic development	5.55E-12	2.91E-10	2.34E-10	144
BP	GO:0031396	regulation of protein ubiquitination	5.66E-12	2.94E-10	2.37E-10	91
BP	GO:0017038	protein import	5.82E-12	3.00E-10	2.41E-10	87
BP	GO:0032386	regulation of intracellular transport	5.97E-12	3.05E-10	2.46E-10	143
BP	GO:0007059	chromosome segregation	6.33E-12	3.21E-10	2.58E-10	128
BP	GO:0006476	protein deacetylation	6.41E-12	3.22E-10	2.59E-10	52

BP	GO:0051052	regulation of DNA metabolic process	7.25E-12	3.62E-10	2.91E-10	137
BP	GO:0006914	autophagy	7.39E-12	3.63E-10	2.92E-10	180
BP	GO:0061919	process utilizing autophagic mechanism	7.39E-12	3.63E-10	2.92E-10	180
BP	GO:0006283	transcription-coupled nucleotide-excision repair	8.27E-12	4.03E-10	3.24E-10	44
BP	GO:0018022	peptidyl-lysine methylation	8.93E-12	4.32E-10	3.47E-10	65
BP	GO:0016574	histone ubiquitination	9.19E-12	4.36E-10	3.51E-10	32
BP	GO:0044766	multi-organism transport	9.23E-12	4.36E-10	3.51E-10	39
BP	GO:1902579	multi-organism localization	9.23E-12	4.36E-10	3.51E-10	39
BP	GO:0045787	positive regulation of cell cycle	1.03E-11	4.82E-10	3.88E-10	148
BP	GO:0006888	endoplasmic reticulum to Golgi vesicle-mediated transport	1.06E-11	4.91E-10	3.95E-10	93
BP	GO:0032508	DNA duplex unwinding	1.06E-11	4.91E-10	3.95E-10	58
BP	GO:0016072	rRNA metabolic process	1.30E-11	5.98E-10	4.81E-10	97
BP	GO:0006401	RNA catabolic process	1.37E-11	6.20E-10	4.99E-10	150
BP	GO:0031123	RNA 3'-end processing	1.37E-11	6.20E-10	4.99E-10	72
BP	GO:0006402	mRNA catabolic process	1.50E-11	6.73E-10	5.42E-10	140
BP	GO:0051225	spindle assembly	1.52E-11	6.76E-10	5.44E-10	57
BP	GO:0032786	positive regulation of DNA-templated transcription, elongation	1.66E-11	7.34E-10	5.91E-10	25
BP	GO:0035601	protein deacylation	1.88E-11	8.24E-10	6.63E-10	55

BP	GO:0061013	regulation of mRNA catabolic process	2.23E-11	9.74E-10	7.84E-10	88
BP	GO:0034605	cellular response to heat	2.43E-11	1.05E-09	8.47E-10	67
BP	GO:0006289	nucleotide-excision repair	2.47E-11	1.06E-09	8.56E-10	57
BP	GO:0098732	macromolecule deacylation	3.10E-11	1.32E-09	1.06E-09	55
BP	GO:0048524	positive regulation of viral process	3.54E-11	1.50E-09	1.21E-09	56
BP	GO:0009411	response to UV	3.85E-11	1.62E-09	1.31E-09	68
BP	GO:0098727	maintenance of cell number	4.60E-11	1.93E-09	1.55E-09	74
BP	GO:0006275	regulation of DNA replication	5.71E-11	2.37E-09	1.91E-09	56
BP	GO:0019827	stem cell population maintenance	6.56E-11	2.71E-09	2.18E-09	73
BP	GO:0051650	establishment of vesicle localization	6.61E-11	2.71E-09	2.18E-09	82
BP	GO:0032200	telomere organization	6.77E-11	2.76E-09	2.22E-09	79
BP	GO:0034976	response to endoplasmic reticulum stress	7.11E-11	2.88E-09	2.32E-09	114
BP	GO:0031398	positive regulation of protein ubiquitination	7.72E-11	3.10E-09	2.50E-09	59
BP	GO:0007051	spindle organization	9.65E-11	3.85E-09	3.10E-09	77
BP	GO:0016575	histone deacetylation	1.00E-10	3.98E-09	3.21E-09	44
BP	GO:0032784	regulation of DNA-templated transcription, elongation	1.04E-10	4.10E-09	3.30E-09	33
BP	GO:0140014	mitotic nuclear division	1.08E-10	4.25E-09	3.42E-09	107
BP	GO:0006302	double-strand break repair	1.09E-10	4.25E-09	3.42E-09	102

BP	GO:0043967	histone H4 acetylation	1.11E-10	4.28E-09	3.44E-09	40
BP	GO:0051298	centrosome duplication	1.11E-10	4.28E-09	3.44E-09	40
BP	GO:0046822	regulation of nucleocytoplasmic transport	1.17E-10	4.47E-09	3.60E-09	54
BP	GO:0006364	rRNA processing	1.35E-10	5.13E-09	4.13E-09	91
BP	GO:0006900	vesicle budding from membrane	1.67E-10	6.32E-09	5.09E-09	53
BP	GO:0010506	regulation of autophagy	1.70E-10	6.40E-09	5.15E-09	126
BP	GO:0006513	protein monoubiquitination	2.92E-10	1.09E-08	8.78E-09	39
BP	GO:0042176	regulation of protein catabolic process	3.01E-10	1.12E-08	8.99E-09	141
BP	GO:0061136	regulation of proteasomal protein catabolic process	3.52E-10	1.29E-08	1.04E-08	80
BP	GO:0090068	positive regulation of cell cycle process	3.53E-10	1.29E-08	1.04E-08	116
BP	GO:0031058	positive regulation of histone modification	3.75E-10	1.37E-08	1.10E-08	48
BP	GO:0048194	Golgi vesicle budding	5.44E-10	1.97E-08	1.59E-08	44
BP	GO:0006353	DNA-templated transcription, termination	6.31E-10	2.27E-08	1.83E-08	42
BP	GO:0034243	regulation of transcription elongation from RNA polymerase II promoter	6.91E-10	2.47E-08	1.99E-08	23
BP	GO:1902850	microtubule cytoskeleton organization involved in mitosis	7.24E-10	2.58E-08	2.08E-08	62
BP	GO:0071824	protein-DNA complex subunit organization	7.55E-10	2.67E-08	2.15E-08	112
BP	GO:0006890	retrograde vesicle-mediated transport, Golgi to endoplasmic reticulum	7.64E-10	2.69E-08	2.17E-08	46
BP	GO:2000058	regulation of ubiquitin-dependent protein catabolic process	7.92E-10	2.78E-08	2.23E-08	68

BP	GO:0016236	macroautophagy	8.28E-10	2.88E-08	2.32E-08	114
BP	GO:1903050	regulation of proteolysis involved in cellular protein catabolic process	8.45E-10	2.91E-08	2.34E-08	89
BP	GO:2001020	regulation of response to DNA damage stimulus	8.45E-10	2.91E-08	2.34E-08	89
BP	GO:0043487	regulation of RNA stability	9.32E-10	3.19E-08	2.57E-08	79
BP	GO:0043488	regulation of mRNA stability	9.86E-10	3.36E-08	2.70E-08	77
BP	GO:0044774	mitotic DNA integrity checkpoint	1.04E-09	3.52E-08	2.83E-08	53
BP	GO:0035196	production of miRNAs involved in gene silencing by miRNA	1.14E-09	3.85E-08	3.10E-08	30
BP	GO:0051568	histone H3-K4 methylation	1.48E-09	4.95E-08	3.99E-08	34
BP	GO:0006892	post-Golgi vesicle-mediated transport	1.49E-09	4.96E-08	4.00E-08	52
BP	GO:1902115	regulation of organelle assembly	1.54E-09	5.09E-08	4.10E-08	82
BP	GO:0042770	signal transduction in response to DNA damage	1.54E-09	5.10E-08	4.10E-08	62
BP	GO:0045930	negative regulation of mitotic cell cycle	1.56E-09	5.13E-08	4.13E-08	126
BP	GO:0032434	regulation of proteasomal ubiquitin-dependent protein catabolic process	2.05E-09	6.68E-08	5.38E-08	58
BP	GO:2001251	negative regulation of chromosome organization	2.27E-09	7.37E-08	5.94E-08	66
BP	GO:0051648	vesicle localization	2.51E-09	8.10E-08	6.52E-08	84
BP	GO:0044773	mitotic DNA damage checkpoint	2.82E-09	9.06E-08	7.29E-08	49
BP	GO:0010508	positive regulation of autophagy	2.93E-09	9.36E-08	7.53E-08	57
BP	GO:0030522	intracellular receptor signaling pathway	3.51E-09	1.12E-07	9.00E-08	107

BP	GO:0032204	regulation of telomere maintenance	3.69E-09	1.17E-07	9.39E-08	43
BP	GO:0098813	nuclear chromosome segregation	3.98E-09	1.25E-07	1.01E-07	102
BP	GO:0030518	intracellular steroid hormone receptor signaling pathway	4.54E-09	1.42E-07	1.14E-07	60
BP	GO:0090316	positive regulation of intracellular protein transport	4.92E-09	1.53E-07	1.23E-07	75
BP	GO:0061014	positive regulation of mRNA catabolic process	5.13E-09	1.59E-07	1.28E-07	30
BP	GO:0032968	positive regulation of transcription elongation from RNA polymerase II promoter	5.49E-09	1.69E-07	1.36E-07	16
BP	GO:0000819	sister chromatid segregation	5.60E-09	1.72E-07	1.38E-07	79
BP	GO:1903362	regulation of cellular protein catabolic process	5.63E-09	1.72E-07	1.39E-07	97
BP	GO:0090307	mitotic spindle assembly	7.51E-09	2.27E-07	1.82E-07	33
BP	GO:0031050	dsRNA processing	7.52E-09	2.27E-07	1.82E-07	31
BP	GO:0070918	production of small RNA involved in gene silencing by RNA	7.52E-09	2.27E-07	1.82E-07	31
BP	GO:0007093	mitotic cell cycle checkpoint	7.64E-09	2.29E-07	1.84E-07	71
BP	GO:0032388	positive regulation of intracellular transport	8.38E-09	2.50E-07	2.01E-07	91
BP	GO:0007099	centriole replication	9.48E-09	2.82E-07	2.27E-07	25
BP	GO:0090329	regulation of DNA-dependent DNA replication	1.05E-08	3.09E-07	2.49E-07	32
BP	GO:0007050	cell cycle arrest	1.21E-08	3.55E-07	2.85E-07	93
BP	GO:0006901	vesicle coating	1.31E-08	3.84E-07	3.09E-07	38
BP	GO:0019058	viral life cycle	1.35E-08	3.92E-07	3.15E-07	120

BP	GO:0030330	DNA damage response, signal transduction by p53 class mediator	1.69E-08	4.91E-07	3.95E-07	51
BP	GO:0090305	nucleic acid phosphodiester bond hydrolysis	1.73E-08	4.99E-07	4.02E-07	112
BP	GO:0006903	vesicle targeting	1.99E-08	5.72E-07	4.61E-07	46
BP	GO:0006417	regulation of translation	2.04E-08	5.83E-07	4.69E-07	147
BP	GO:0071478	cellular response to radiation	2.37E-08	6.76E-07	5.44E-07	78
BP	GO:0048199	vesicle targeting, to, from or within Golgi	2.54E-08	7.18E-07	5.78E-07	39
BP	GO:0032201	telomere maintenance via semi-conservative replication	2.55E-08	7.19E-07	5.79E-07	20
BP	GO:0043044	ATP-dependent chromatin remodeling	2.66E-08	7.47E-07	6.02E-07	44
BP	GO:2000104	negative regulation of DNA-dependent DNA replication	2.85E-08	7.97E-07	6.41E-07	19
BP	GO:0000070	mitotic sister chromatid segregation	3.18E-08	8.84E-07	7.12E-07	65
BP	GO:0070936	protein K48-linked ubiquitination	3.55E-08	9.83E-07	7.92E-07	32
BP	GO:0090114	COPII-coated vesicle budding	3.63E-08	1.00E-06	8.06E-07	38
BP	GO:0006310	DNA recombination	3.65E-08	1.00E-06	8.06E-07	108
BP	GO:0006360	transcription by RNA polymerase I	3.80E-08	1.04E-06	8.37E-07	35
BP	GO:0000280	nuclear division	4.16E-08	1.13E-06	9.11E-07	141
BP	GO:0030521	androgen receptor signaling pathway	4.45E-08	1.21E-06	9.71E-07	33
BP	GO:1903829	positive regulation of cellular protein localization	4.67E-08	1.26E-06	1.02E-06	117
BP	GO:0031062	positive regulation of histone methylation	4.72E-08	1.27E-06	1.02E-06	25

BP	GO:0006470	protein dephosphorylation	5.09E-08	1.36E-06	1.10E-06	116
BP	GO:0034248	regulation of cellular amide metabolic process	5.25E-08	1.40E-06	1.13E-06	160
BP	GO:0048285	organelle fission	6.79E-08	1.80E-06	1.45E-06	152
BP	GO:0031440	regulation of mRNA 3'-end processing	6.99E-08	1.85E-06	1.49E-06	20
BP	GO:0021915	neural tube development	7.20E-08	1.89E-06	1.52E-06	67
BP	GO:0006446	regulation of translational initiation	7.24E-08	1.89E-06	1.52E-06	40
BP	GO:1903312	negative regulation of mRNA metabolic process	7.24E-08	1.89E-06	1.52E-06	40
BP	GO:0009408	response to heat	7.43E-08	1.93E-06	1.55E-06	72
BP	GO:0010833	telomere maintenance via telomere lengthening	7.86E-08	2.03E-06	1.64E-06	41
BP	GO:0070507	regulation of microtubule cytoskeleton organization	8.19E-08	2.11E-06	1.70E-06	75
BP	GO:0071479	cellular response to ionizing radiation	8.46E-08	2.17E-06	1.75E-06	37
BP	GO:0071897	DNA biosynthetic process	8.80E-08	2.25E-06	1.81E-06	78
BP	GO:0030705	cytoskeleton-dependent intracellular transport	9.20E-08	2.34E-06	1.89E-06	73
BP	GO:0009416	response to light stimulus	9.70E-08	2.46E-06	1.98E-06	113
BP	GO:0008156	negative regulation of DNA replication	9.83E-08	2.47E-06	1.99E-06	25
BP	GO:0098534	centriole assembly	9.83E-08	2.47E-06	1.99E-06	25
BP	GO:0072698	protein localization to microtubule cytoskeleton	9.90E-08	2.48E-06	2.00E-06	29
BP	GO:0009048	dosage compensation by inactivation of X chromosome	1.01E-07	2.51E-06	2.02E-06	15

BP	GO:0007265	Ras protein signal transduction	1.02E-07	2.54E-06	2.04E-06	151
BP	GO:0048207	vesicle targeting, rough ER to cis-Golgi	1.07E-07	2.63E-06	2.11E-06	35
BP	GO:0048208	COPII vesicle coating	1.07E-07	2.63E-06	2.11E-06	35
BP	GO:0016311	dephosphorylation	1.24E-07	3.04E-06	2.45E-06	159
BP	GO:0048511	rhythmic process	1.34E-07	3.27E-06	2.63E-06	107
BP	GO:0043687	post-translational protein modification	1.34E-07	3.27E-06	2.63E-06	126
BP	GO:0051090	regulation of DNA-binding transcription factor activity	1.38E-07	3.34E-06	2.69E-06	146
BP	GO:0061647	histone H3-K9 modification	1.39E-07	3.36E-06	2.70E-06	28
BP	GO:1901991	negative regulation of mitotic cell cycle phase transition	1.52E-07	3.65E-06	2.94E-06	93
BP	GO:0046782	regulation of viral transcription	1.53E-07	3.66E-06	2.95E-06	34
BP	GO:0060964	regulation of gene silencing by miRNA	1.55E-07	3.69E-06	2.97E-06	51
BP	GO:0043401	steroid hormone mediated signaling pathway	1.56E-07	3.70E-06	2.98E-06	73
BP	GO:0001510	RNA methylation	1.74E-07	4.12E-06	3.32E-06	40
BP	GO:0035966	response to topologically incorrect protein	1.86E-07	4.39E-06	3.53E-06	78
BP	GO:0035303	regulation of dephosphorylation	1.91E-07	4.50E-06	3.62E-06	81
BP	GO:0050434	positive regulation of viral transcription	1.97E-07	4.61E-06	3.71E-06	25
BP	GO:0071826	ribonucleoprotein complex subunit organization	2.08E-07	4.85E-06	3.90E-06	89
BP	GO:0060147	regulation of posttranscriptional gene silencing	2.15E-07	4.98E-06	4.01E-06	52

BP	GO:0060966	regulation of gene silencing by RNA	2.15E-07	4.98E-06	4.01E-06	52
BP	GO:0042752	regulation of circadian rhythm	2.18E-07	5.03E-06	4.05E-06	51
BP	GO:1904356	regulation of telomere maintenance via telomere lengthening	2.19E-07	5.03E-06	4.05E-06	33
BP	GO:0060968	regulation of gene silencing	2.43E-07	5.56E-06	4.48E-06	59
BP	GO:0016241	regulation of macroautophagy	2.57E-07	5.87E-06	4.73E-06	69
BP	GO:0006378	mRNA polyadenylation	2.72E-07	6.20E-06	4.99E-06	26
BP	GO:0007052	mitotic spindle organization	3.14E-07	7.11E-06	5.72E-06	48
BP	GO:1905508	protein localization to microtubule organizing center	3.17E-07	7.15E-06	5.76E-06	21
BP	GO:0071214	cellular response to abiotic stimulus	3.28E-07	7.32E-06	5.89E-06	116
BP	GO:0104004	cellular response to environmental stimulus	3.28E-07	7.32E-06	5.89E-06	116
BP	GO:0007623	circadian rhythm	3.29E-07	7.32E-06	5.89E-06	80
BP	GO:0050792	regulation of viral process	3.29E-07	7.32E-06	5.89E-06	80
BP	GO:0043984	histone H4-K16 acetylation	3.53E-07	7.83E-06	6.31E-06	16
BP	GO:0051567	histone H3-K9 methylation	3.70E-07	8.19E-06	6.59E-06	23
BP	GO:0031057	negative regulation of histone modification	3.80E-07	8.38E-06	6.74E-06	25
BP	GO:0043903	regulation of interspecies interactions between organisms	3.82E-07	8.39E-06	6.76E-06	84
BP	GO:0034502	protein localization to chromosome	3.98E-07	8.70E-06	7.00E-06	40
BP	GO:0006986	response to unfolded protein	4.05E-07	8.83E-06	7.11E-06	70

BP	GO:0061157	mRNA destabilization	4.14E-07	9.00E-06	7.25E-06	20
BP	GO:0006661	phosphatidylinositol biosynthetic process	4.23E-07	9.16E-06	7.37E-06	51
BP	GO:0031647	regulation of protein stability	4.46E-07	9.62E-06	7.75E-06	102
BP	GO:0046605	regulation of centrosome cycle	4.49E-07	9.64E-06	7.76E-06	31
BP	GO:1901988	negative regulation of cell cycle phase transition	4.64E-07	9.94E-06	8.00E-06	97
BP	GO:0000725	recombinational repair	4.76E-07	1.02E-05	8.18E-06	57
BP	GO:0006369	termination of RNA polymerase II transcription	5.04E-07	1.07E-05	8.64E-06	22
BP	GO:1903902	positive regulation of viral life cycle	5.14E-07	1.09E-05	8.78E-06	32
BP	GO:0050686	negative regulation of mRNA processing	5.35E-07	1.13E-05	9.10E-06	19
BP	GO:0035967	cellular response to topologically incorrect protein	5.52E-07	1.16E-05	9.35E-06	65
BP	GO:0018105	peptidyl-serine phosphorylation	5.53E-07	1.16E-05	9.35E-06	106
BP	GO:0044783	G1 DNA damage checkpoint	5.77E-07	1.21E-05	9.72E-06	33
BP	GO:0065004	protein-DNA complex assembly	6.29E-07	1.31E-05	1.06E-05	91
BP	GO:0007004	telomere maintenance via telomerase	6.91E-07	1.43E-05	1.16E-05	35
BP	GO:0035329	hippo signaling	7.34E-07	1.52E-05	1.22E-05	23
BP	GO:0072401	signal transduction involved in DNA integrity checkpoint	7.39E-07	1.52E-05	1.22E-05	36
BP	GO:0072422	signal transduction involved in DNA damage checkpoint	7.39E-07	1.52E-05	1.22E-05	36
BP	GO:0030968	endoplasmic reticulum unfolded protein response	7.75E-07	1.59E-05	1.28E-05	52

BP	GO:0032922	circadian regulation of gene expression	8.29E-07	1.69E-05	1.36E-05	32
BP	GO:0046824	positive regulation of nucleocytoplasmic transport	8.29E-07	1.69E-05	1.36E-05	32
BP	GO:0033523	histone H2B ubiquitination	8.76E-07	1.78E-05	1.43E-05	11
BP	GO:0000724	double-strand break repair via homologous recombination	8.91E-07	1.80E-05	1.45E-05	56
BP	GO:0043631	RNA polyadenylation	9.07E-07	1.83E-05	1.47E-05	26
BP	GO:0071539	protein localization to centrosome	9.15E-07	1.84E-05	1.48E-05	20
BP	GO:0051098	regulation of binding	9.98E-07	2.00E-05	1.61E-05	126
BP	GO:0032205	negative regulation of telomere maintenance	1.01E-06	2.03E-05	1.63E-05	22
BP	GO:0072395	signal transduction involved in cell cycle checkpoint	1.12E-06	2.24E-05	1.80E-05	36
BP	GO:0000288	nuclear-transcribed mRNA catabolic process, deadenylation-dependent decay	1.17E-06	2.33E-05	1.88E-05	37
BP	GO:0007549	dosage compensation	1.18E-06	2.34E-05	1.88E-05	15
BP	GO:0010390	histone monoubiquitination	1.22E-06	2.40E-05	1.93E-05	19
BP	GO:0071383	cellular response to steroid hormone stimulus	1.25E-06	2.46E-05	1.98E-05	90
BP	GO:0018023	peptidyl-lysine trimethylation	1.29E-06	2.52E-05	2.03E-05	25
BP	GO:0031571	mitotic G1 DNA damage checkpoint	1.32E-06	2.56E-05	2.06E-05	32
BP	GO:0044819	mitotic G1/S transition checkpoint	1.32E-06	2.56E-05	2.06E-05	32
BP	GO:0006998	nuclear envelope organization	1.32E-06	2.56E-05	2.06E-05	28
BP	GO:0006333	chromatin assembly or disassembly	1.38E-06	2.67E-05	2.15E-05	84

BP	GO:0030488	tRNA methylation	1.40E-06	2.70E-05	2.18E-05	23
BP	GO:0032886	regulation of microtubule-based process	1.43E-06	2.76E-05	2.22E-05	81
BP	GO:0044380	protein localization to cytoskeleton	1.52E-06	2.92E-05	2.35E-05	29
BP	GO:0045023	G0 to G1 transition	1.59E-06	3.03E-05	2.44E-05	26
BP	GO:0051569	regulation of histone H3-K4 methylation	1.60E-06	3.05E-05	2.45E-05	18
BP	GO:0022618	ribonucleoprotein complex assembly	1.70E-06	3.22E-05	2.59E-05	84
BP	GO:0001841	neural tube formation	1.73E-06	3.27E-05	2.63E-05	46
BP	GO:0016197	endosomal transport	1.84E-06	3.48E-05	2.80E-05	83
BP	GO:0050779	RNA destabilization	1.91E-06	3.60E-05	2.90E-05	20
BP	GO:0034620	cellular response to unfolded protein	1.95E-06	3.66E-05	2.95E-05	57
BP	GO:0033522	histone H2A ubiquitination	2.07E-06	3.87E-05	3.12E-05	17
BP	GO:0070316	regulation of G0 to G1 transition	2.26E-06	4.22E-05	3.40E-05	25
BP	GO:0010332	response to gamma radiation	2.46E-06	4.57E-05	3.68E-05	29
BP	GO:1905515	non-motile cilium assembly	2.46E-06	4.57E-05	3.68E-05	29
BP	GO:0036503	ERAD pathway	2.50E-06	4.62E-05	3.72E-05	44
BP	GO:0071158	positive regulation of cell cycle arrest	2.59E-06	4.78E-05	3.85E-05	38
BP	GO:1904837	beta-catenin-TCF complex assembly	2.60E-06	4.78E-05	3.85E-05	19
BP	GO:0018209	peptidyl-serine modification	2.63E-06	4.82E-05	3.88E-05	110

BP	GO:0006282	regulation of DNA repair	2.66E-06	4.86E-05	3.91E-05	51
BP	GO:0072413	signal transduction involved in mitotic cell cycle checkpoint	2.72E-06	4.93E-05	3.97E-05	30
BP	GO:1902402	signal transduction involved in mitotic DNA damage checkpoint	2.72E-06	4.93E-05	3.97E-05	30
BP	GO:1902403	signal transduction involved in mitotic DNA integrity checkpoint	2.72E-06	4.93E-05	3.97E-05	30
BP	GO:0010970	transport along microtubule	2.87E-06	5.19E-05	4.18E-05	63
BP	GO:0051054	positive regulation of DNA metabolic process	2.94E-06	5.30E-05	4.27E-05	72
BP	GO:0016239	positive regulation of macroautophagy	3.33E-06	5.98E-05	4.82E-05	33
BP	GO:0010921	regulation of phosphatase activity	3.45E-06	6.18E-05	4.98E-05	67
BP	GO:0032210	regulation of telomere maintenance via telomerase	3.55E-06	6.33E-05	5.10E-05	28
BP	GO:0046825	regulation of protein export from nucleus	3.65E-06	6.49E-05	5.23E-05	22
BP	GO:0006303	double-strand break repair via nonhomologous end joining	3.73E-06	6.63E-05	5.34E-05	39
BP	GO:0072431	signal transduction involved in mitotic G1 DNA damage checkpoint	3.92E-06	6.92E-05	5.57E-05	29
BP	GO:1902400	intracellular signal transduction involved in G1 DNA damage checkpoint	3.92E-06	6.92E-05	5.57E-05	29
BP	GO:0051091	positive regulation of DNA-binding transcription factor activity	3.95E-06	6.96E-05	5.60E-05	92
BP	GO:0043966	histone H3 acetylation	4.25E-06	7.47E-05	6.01E-05	30
BP	GO:0046831	regulation of RNA export from nucleus	4.50E-06	7.88E-05	6.34E-05	11
BP	GO:0006893	Golgi to plasma membrane transport	4.54E-06	7.93E-05	6.39E-05	31
BP	GO:0045005	DNA-dependent DNA replication maintenance of fidelity	4.60E-06	7.99E-05	6.43E-05	23

BP	GO:0070317	negative regulation of G0 to G1 transition	4.60E-06	7.99E-05	6.43E-05	23
BP	GO:0009451	RNA modification	4.64E-06	8.04E-05	6.47E-05	63
BP	GO:1904353	regulation of telomere capping	4.69E-06	8.10E-05	6.52E-05	17
BP	GO:0032239	regulation of nucleobase-containing compound transport	4.73E-06	8.14E-05	6.56E-05	12
BP	GO:0051865	protein autoubiquitination	4.98E-06	8.56E-05	6.89E-05	33
BP	GO:0006301	postreplication repair	5.10E-06	8.74E-05	7.04E-05	27
BP	GO:0006482	protein demethylation	5.27E-06	8.93E-05	7.19E-05	19
BP	GO:0008214	protein dealkylation	5.27E-06	8.93E-05	7.19E-05	19
BP	GO:0010165	response to X-ray	5.27E-06	8.93E-05	7.19E-05	19
BP	GO:0043470	regulation of carbohydrate catabolic process	5.27E-06	8.93E-05	7.19E-05	39
BP	GO:0070897	transcription preinitiation complex assembly	5.56E-06	9.39E-05	7.56E-05	24
BP	GO:0022616	DNA strand elongation	6.19E-06	0.0001043	8.40E-05	16
BP	GO:2000736	regulation of stem cell differentiation	7.22E-06	0.0001208	9.72E-05	48
BP	GO:0018196	peptidyl-asparagine modification	7.25E-06	0.0001208	9.72E-05	20
BP	GO:0033120	positive regulation of RNA splicing	7.25E-06	0.0001208	9.72E-05	20
BP	GO:0016577	histone demethylation	7.27E-06	0.0001208	9.72E-05	18
BP	GO:0031365	N-terminal protein amino acid modification	7.27E-06	0.0001208	9.72E-05	18
BP	GO:0030099	myeloid cell differentiation	7.81E-06	0.0001296	0.000104301	134

BP	GO:0046599	regulation of centriole replication	8.04E-06	0.0001328	0.000106945	15
BP	GO:0018107	peptidyl-threonine phosphorylation	8.05E-06	0.0001328	0.000106945	51
BP	GO:0051099	positive regulation of binding	8.42E-06	0.0001385	0.000111515	67
BP	GO:1901800	positive regulation of proteasomal protein catabolic process	8.69E-06	0.0001425	0.000114755	44
BP	GO:0006977	DNA damage response, signal transduction by p53 class mediator resulting in cell cycle arrest	8.83E-06	0.0001444	0.000116284	28
BP	GO:1903052	positive regulation of proteolysis involved in cellular protein catabolic process	9.03E-06	0.0001474	0.000118695	49
BP	GO:2000060	positive regulation of ubiquitin-dependent protein catabolic process	9.70E-06	0.0001579	0.000127102	41
BP	GO:1905268	negative regulation of chromatin organization	9.90E-06	0.0001607	0.000129402	30
BP	GO:0090169	regulation of spindle assembly	9.95E-06	0.0001612	0.000129748	17
BP	GO:0030218	erythrocyte differentiation	1.01E-05	0.0001625	0.000130867	47
BP	GO:0031061	negative regulation of histone methylation	1.02E-05	0.0001647	0.000132582	14
BP	GO:0006278	RNA-dependent DNA biosynthetic process	1.08E-05	0.0001742	0.000140239	35
BP	GO:0071156	regulation of cell cycle arrest	1.11E-05	0.0001783	0.000143543	45
BP	GO:0010639	negative regulation of organelle organization	1.12E-05	0.0001793	0.000144361	127
BP	GO:0019080	viral gene expression	1.24E-05	0.000197	0.000158603	70
BP	GO:0007088	regulation of mitotic nuclear division	1.25E-05	0.000199	0.000160194	62
BP	GO:0010824	regulation of centrosome duplication	1.34E-05	0.0002127	0.000171217	23
BP	GO:0001672	regulation of chromatin assembly or disassembly	1.35E-05	0.000214	0.000172308	16

BP	GO:0010998	regulation of translational initiation by eIF2 alpha phosphorylation	1.40E-05	0.0002204	0.000177437	9
BP	GO:0001843	neural tube closure	1.41E-05	0.0002223	0.000178951	39
BP	GO:0036124	histone H3-K9 trimethylation	1.49E-05	0.0002339	0.000188347	12
BP	GO:0080182	histone H3-K4 trimethylation	1.49E-05	0.0002339	0.000188347	12
BP	GO:0006984	ER-nucleus signaling pathway	1.53E-05	0.0002378	0.000191462	24
BP	GO:0030490	maturation of SSU-rRNA	1.53E-05	0.0002378	0.000191462	24
BP	GO:0034101	erythrocyte homeostasis	1.54E-05	0.0002394	0.000192769	49
BP	GO:1902117	positive regulation of organelle assembly	1.55E-05	0.0002399	0.000193108	34
BP	GO:0071559	response to transforming growth factor beta	1.63E-05	0.0002514	0.000202383	88
BP	GO:0006999	nuclear pore organization	1.66E-05	0.0002562	0.000206303	11
BP	GO:0014020	primary neural tube formation	1.78E-05	0.0002745	0.000221021	41
BP	GO:0006297	nucleotide-excision repair, DNA gap filling	1.82E-05	0.0002794	0.000224975	15
BP	GO:0000726	non-recombinational repair	1.86E-05	0.0002845	0.000229016	40
BP	GO:0018279	protein N-linked glycosylation via asparagine	1.88E-05	0.0002871	0.000231116	19
BP	GO:0040001	establishment of mitotic spindle localization	1.88E-05	0.0002871	0.000231116	19
BP	GO:0000956	nuclear-transcribed mRNA catabolic process	1.93E-05	0.0002919	0.000234975	74
BP	GO:0060606	tube closure	1.93E-05	0.0002919	0.000234975	39
BP	GO:0090224	regulation of spindle organization	1.93E-05	0.0002919	0.000234975	22

BP	GO:0070076	histone lysine demethylation	1.99E-05	0.0003005	0.000241936	17
BP	GO:0034644	cellular response to UV	2.11E-05	0.0003174	0.000255503	36
BP	GO:0050000	chromosome localization	2.19E-05	0.0003283	0.000264316	34
BP	GO:0008033	tRNA processing	2.24E-05	0.000336	0.000270516	51
BP	GO:0009266	response to temperature stimulus	2.33E-05	0.0003485	0.000280592	84
BP	GO:0061614	pri-miRNA transcription by RNA polymerase II	2.44E-05	0.0003642	0.000293175	24
BP	GO:0032608	interferon-beta production	2.65E-05	0.0003941	0.000317259	25
BP	GO:0018210	peptidyl-threonine modification	2.66E-05	0.0003952	0.000318141	52
BP	GO:0050685	positive regulation of mRNA processing	2.67E-05	0.0003955	0.000318453	18
BP	GO:0007020	microtubule nucleation	2.77E-05	0.0004086	0.000328985	16
BP	GO:0006400	tRNA modification	2.81E-05	0.0004145	0.000333717	37
BP	GO:0000186	activation of MAPKK activity	2.82E-05	0.0004145	0.000333717	26
BP	GO:0007179	transforming growth factor beta receptor signaling pathway	3.03E-05	0.0004438	0.000357305	71
BP	GO:2000756	regulation of peptidyl-lysine acetylation	3.05E-05	0.0004462	0.000359249	28
BP	GO:0006110	regulation of glycolytic process	3.06E-05	0.0004462	0.000359249	34
BP	GO:0001832	blastocyst growth	3.15E-05	0.0004591	0.000369615	13
BP	GO:0006284	base-excision repair	3.17E-05	0.0004613	0.000371429	22
BP	GO:0051817	modulation of process of other organism involved in symbiotic interaction	3.18E-05	0.0004617	0.000371689	41

BP	GO:0098876	vesicle-mediated transport to the plasma membrane	3.35E-05	0.0004841	0.000389735	40
BP	GO:0030177	positive regulation of Wnt signaling pathway	3.45E-05	0.0004984	0.000401279	65
BP	GO:0071560	cellular response to transforming growth factor beta stimulus	3.53E-05	0.0005079	0.000408886	85
BP	GO:0045732	positive regulation of protein catabolic process	3.69E-05	0.0005295	0.000426275	75
BP	GO:0008654	phospholipid biosynthetic process	3.72E-05	0.0005328	0.000428929	88
BP	GO:0031330	negative regulation of cellular catabolic process	3.79E-05	0.0005426	0.000436811	86
BP	GO:0032648	regulation of interferon-beta production	3.82E-05	0.0005456	0.000439261	24
BP	GO:0097193	intrinsic apoptotic signaling pathway	3.90E-05	0.000555	0.000446813	96
BP	GO:0060236	regulation of mitotic spindle organization	4.00E-05	0.0005671	0.00045653	20
BP	GO:0006474	N-terminal protein amino acid acetylation	4.01E-05	0.0005671	0.00045653	12
BP	GO:0043968	histone H2A acetylation	4.01E-05	0.0005671	0.00045653	12
BP	GO:0000731	DNA synthesis involved in DNA repair	4.07E-05	0.0005718	0.00046035	25
BP	GO:0032206	positive regulation of telomere maintenance	4.07E-05	0.0005718	0.00046035	25
BP	GO:0048863	stem cell differentiation	4.07E-05	0.0005718	0.00046035	87
BP	GO:0032436	positive regulation of proteasomal ubiquitin-dependent protein catabolic process	4.11E-05	0.0005754	0.000463251	35
BP	GO:0070498	interleukin-1-mediated signaling pathway	4.21E-05	0.0005885	0.00047383	41
BP	GO:0030433	ubiquitin-dependent ERAD pathway	4.23E-05	0.0005902	0.00047516	34
BP	GO:0031098	stress-activated protein kinase signaling cascade	4.29E-05	0.0005961	0.000479891	103

BP	GO:0099111	microtubule-based transport	4.29E-05	0.0005961	0.000479891	67
BP	GO:0007033	vacuole organization	4.30E-05	0.0005963	0.000480063	60
BP	GO:0051303	establishment of chromosome localization	4.34E-05	0.0005999	0.000482946	33
BP	GO:0006323	DNA packaging	4.55E-05	0.0006279	0.000505522	84
BP	GO:0009755	hormone-mediated signaling pathway	4.70E-05	0.0006471	0.000520992	80
BP	GO:0006356	regulation of transcription by RNA polymerase I	4.80E-05	0.0006586	0.000530273	18
BP	GO:1903364	positive regulation of cellular protein catabolic process	4.94E-05	0.0006772	0.000545229	53
BP	GO:0043122	regulation of I-kappaB kinase/NF-kappaB signaling	5.07E-05	0.0006938	0.000558539	81
BP	GO:0051783	regulation of nuclear division	5.19E-05	0.0007082	0.000570166	67
BP	GO:0000466	maturation of 5.8S rRNA from tricistronic rRNA transcript (SSU-rRNA, 5.8S rRNA, LSU-rRNA)	5.24E-05	0.0007083	0.000570224	14
BP	GO:0000469	cleavage involved in rRNA processing	5.24E-05	0.0007083	0.000570224	14
BP	GO:0045943	positive regulation of transcription by RNA polymerase I	5.24E-05	0.0007083	0.000570224	14
BP	GO:1901522	positive regulation of transcription from RNA polymerase II promoter involved in cellular response to chemical stimulus	5.24E-05	0.0007083	0.000570224	14
BP	GO:0034067	protein localization to Golgi apparatus	5.34E-05	0.0007193	0.00057907	16
BP	GO:1904357	negative regulation of telomere maintenance via telomere lengthening	5.34E-05	0.0007193	0.00057907	16
BP	GO:1901532	regulation of hematopoietic progenitor cell differentiation	5.38E-05	0.0007224	0.000581614	36
BP	GO:0045070	positive regulation of viral genome replication	5.76E-05	0.0007717	0.000621273	19
BP	GO:0035305	negative regulation of dephosphorylation	5.94E-05	0.0007952	0.000640207	44

BP	GO:0046607	positive regulation of centrosome cycle	6.10E-05	0.0008143	0.000655612	9
BP	GO:1901983	regulation of protein acetylation	6.16E-05	0.0008205	0.000660597	32
BP	GO:0031146	SCF-dependent proteasomal ubiquitin-dependent protein catabolic process	6.20E-05	0.0008235	0.000663017	39
BP	GO:2000278	regulation of DNA biosynthetic process	6.35E-05	0.0008422	0.00067806	43
BP	GO:0010847	regulation of chromatin assembly	7.11E-05	0.0009387	0.000755714	13
BP	GO:1901673	regulation of mitotic spindle assembly	7.11E-05	0.0009387	0.000755714	13
BP	GO:2000134	negative regulation of G1/S transition of mitotic cell cycle	7.26E-05	0.0009571	0.00077059	48
BP	GO:1902893	regulation of pri-miRNA transcription by RNA polymerase II	7.37E-05	0.0009694	0.000780479	21
BP	GO:0043123	positive regulation of I-kappaB kinase/NF-kappaB signaling	7.47E-05	0.0009806	0.000789509	65
BP	GO:0009895	negative regulation of catabolic process	7.49E-05	0.0009807	0.000789581	100
BP	GO:1990823	response to leukemia inhibitory factor	8.14E-05	0.0010622	0.000855194	39
BP	GO:1990830	cellular response to leukemia inhibitory factor	8.14E-05	0.0010622	0.000855194	39
BP	GO:0006891	intra-Golgi vesicle-mediated transport	8.29E-05	0.0010793	0.000868905	18
BP	GO:0070534	protein K63-linked ubiquitination	8.85E-05	0.0011491	0.000925147	24
BP	GO:0098840	protein transport along microtubule	8.96E-05	0.0011587	0.000932821	30
BP	GO:0099118	microtubule-based protein transport	8.96E-05	0.0011587	0.000932821	30
BP	GO:0035065	regulation of histone acetylation	9.09E-05	0.001169	0.00094113	25
BP	GO:0036297	interstrand cross-link repair	9.09E-05	0.001169	0.00094113	25

BP	GO:0042073	intraciliary transport	9.09E-05	0.001169	0.00094113	25
BP	GO:0051983	regulation of chromosome segregation	9.35E-05	0.0011998	0.000965923	41
BP	GO:0007034	vacuolar transport	9.50E-05	0.0012051	0.000970187	53
BP	GO:0007221	positive regulation of transcription of Notch receptor target	9.51E-05	0.0012051	0.000970187	12
BP	GO:0031293	membrane protein intracellular domain proteolysis	9.51E-05	0.0012051	0.000970187	12
BP	GO:0031445	regulation of heterochromatin assembly	9.51E-05	0.0012051	0.000970187	12
BP	GO:0031468	nuclear envelope reassembly	9.51E-05	0.0012051	0.000970187	12
BP	GO:0043555	regulation of translation in response to stress	9.51E-05	0.0012051	0.000970187	12
BP	GO:0051053	negative regulation of DNA metabolic process	9.88E-05	0.0012491	0.001005658	47
BP	GO:1905897	regulation of response to endoplasmic reticulum stress	0.0001012	0.0012774	0.001028421	35
BP	GO:0016578	histone deubiquitination	0.0001055	0.0013238	0.0010658	14
BP	GO:0035561	regulation of chromatin binding	0.0001055	0.0013238	0.0010658	14
BP	GO:0051570	regulation of histone H3-K9 methylation	0.0001055	0.0013238	0.0010658	14
BP	GO:0002262	myeloid cell homeostasis	0.0001077	0.0013479	0.001085215	54
BP	GO:1903706	regulation of hemopoiesis	0.0001085	0.0013558	0.001091525	144
BP	GO:0006487	protein N-linked glycosylation	0.000111	0.0013842	0.001114395	33
BP	GO:0090501	RNA phosphodiester bond hydrolysis	0.0001113	0.0013852	0.001115224	56
BP	GO:0002831	regulation of response to biotic stimulus	0.000113	0.0014032	0.001129719	124

BP	GO:0033143	regulation of intracellular steroid hormone receptor signaling pathway	0.0001157	0.0014335	0.001154082	32
BP	GO:0006363	termination of RNA polymerase I transcription	0.0001195	0.0014783	0.001190135	17
BP	GO:1903747	regulation of establishment of protein localization to mitochondrion	0.00012	0.0014815	0.001192767	31
BP	GO:0001838	embryonic epithelial tube formation	0.0001241	0.0015284	0.001230467	47
BP	GO:0051571	positive regulation of histone H3-K4 methylation	0.0001248	0.0015315	0.001233007	11
BP	GO:0060628	regulation of ER to Golgi vesicle-mediated transport	0.0001248	0.0015315	0.001233007	11
BP	GO:0051653	spindle localization	0.0001277	0.0015639	0.001259079	23
BP	GO:1903008	organelle disassembly	0.000129	0.0015767	0.001269406	40
BP	GO:1902807	negative regulation of cell cycle G1/S phase transition	0.0001319	0.0016086	0.001295085	49
BP	GO:0035148	tube formation	0.0001321	0.0016086	0.001295085	54
BP	GO:0002183	cytoplasmic translational initiation	0.0001385	0.0016768	0.001349941	18
BP	GO:0009452	7-methylguanosine RNA capping	0.0001385	0.0016768	0.001349941	18
BP	GO:0036260	RNA capping	0.0001385	0.0016768	0.001349941	18
BP	GO:0097581	lamellipodium organization	0.0001417	0.0017125	0.001378723	34
BP	GO:0032606	type I interferon production	0.000143	0.0017247	0.001388535	48
BP	GO:0007249	I-kappaB kinase/NF-kappaB signaling	0.0001463	0.0017601	0.001417071	88
BP	GO:2000737	negative regulation of stem cell differentiation	0.0001473	0.0017692	0.001424379	13
BP	GO:0016358	dendrite development	0.0001491	0.0017878	0.001439314	78

BP	GO:0006270	DNA replication initiation	0.0001546	0.0018463	0.00148647	19
BP	GO:1904358	positive regulation of telomere maintenance via telomere lengthening	0.0001546	0.0018463	0.00148647	19
BP	GO:0043558	regulation of translational initiation in response to stress	0.0001591	0.0018929	0.001523981	10
BP	GO:0090110	COPII-coated vesicle cargo loading	0.0001591	0.0018929	0.001523981	10
BP	GO:0072175	epithelial tube formation	0.0001636	0.0019421	0.001563542	49
BP	GO:0006497	protein lipidation	0.0001669	0.0019775	0.001592079	36
BP	GO:0019083	viral transcription	0.0001693	0.0020025	0.001612164	62
BP	GO:0070988	demethylation	0.0001697	0.0020036	0.001613119	30
BP	GO:0035304	regulation of protein dephosphorylation	0.0001705	0.0020097	0.001618011	51
BP	GO:0000132	establishment of mitotic spindle orientation	0.0001723	0.0020266	0.001631576	16
BP	GO:0051348	negative regulation of transferase activity	0.0001782	0.0020926	0.001684709	92
BP	GO:1902806	regulation of cell cycle G1/S phase transition	0.0001789	0.0020961	0.001687536	69
BP	GO:0030032	lamellipodium assembly	0.0001811	0.002118	0.001705171	28
BP	GO:0046488	phosphatidylinositol metabolic process	0.0001852	0.0021623	0.001740854	61
BP	GO:0045815	positive regulation of gene expression, epigenetic	0.0001887	0.0021911	0.001764078	26
BP	GO:1903749	positive regulation of establishment of protein localization to mitochondrion	0.0001887	0.0021911	0.001764078	26
BP	GO:1904589	regulation of protein import	0.0001887	0.0021911	0.001764078	26
BP	GO:0006383	transcription by RNA polymerase III	0.0001906	0.002204	0.001774432	25

BP	GO:0016233	telomere capping	0.0001906	0.002204	0.001774432	25
BP	GO:0031453	positive regulation of heterochromatin assembly	0.0001939	0.002239	0.001802573	9
BP	GO:0006304	DNA modification	0.0001969	0.0022644	0.001823017	44
BP	GO:0007569	cell aging	0.0001969	0.0022644	0.001823017	44
BP	GO:0034063	stress granule assembly	0.0002041	0.0023333	0.001878546	12
BP	GO:0048025	negative regulation of mRNA splicing, via spliceosome	0.0002041	0.0023333	0.001878546	12
BP	GO:1903798	regulation of production of miRNAs involved in gene silencing by miRNA	0.0002041	0.0023333	0.001878546	12
BP	GO:0051403	stress-activated MAPK cascade	0.0002044	0.0023333	0.001878546	92
BP	GO:0032481	positive regulation of type I interferon production	0.000209	0.0023818	0.001917564	32
BP	GO:0000338	protein deneddylation	0.0002191	0.0024793	0.001996096	8
BP	GO:0033182	regulation of histone ubiquitination	0.0002191	0.0024793	0.001996096	8
BP	GO:0070816	phosphorylation of RNA polymerase II C-terminal domain	0.0002191	0.0024793	0.001996096	8
BP	GO:0090309	positive regulation of DNA methylation-dependent heterochromatin assembly	0.0002191	0.0024793	0.001996096	8
BP	GO:0045088	regulation of innate immune response	0.0002213	0.0024993	0.00201214	97
BP	GO:0000291	nuclear-transcribed mRNA catabolic process, exonucleolytic	0.0002242	0.0025233	0.002031495	18
BP	GO:0032435	negative regulation of proteasomal ubiquitin-dependent protein catabolic process	0.0002242	0.0025233	0.002031495	18
BP	GO:0016482	cytosolic transport	0.0002414	0.0027112	0.002182793	56
BP	GO:2001022	positive regulation of response to DNA damage stimulus	0.0002443	0.0027392	0.002205349	38

BP	GO:0009649	entrainment of circadian clock	0.0002485	0.0027807	0.002238741	15
BP	GO:0019985	translesion synthesis	0.000257	0.0028707	0.002311155	20
BP	GO:0048041	focal adhesion assembly	0.0002616	0.0029178	0.00234911	33
BP	GO:0007080	mitotic metaphase plate congression	0.0002665	0.0029612	0.002384016	21
BP	GO:0090311	regulation of protein deacetylation	0.0002665	0.0029612	0.002384016	21
BP	GO:0045646	regulation of erythrocyte differentiation	0.000272	0.0030173	0.002429243	22
BP	GO:0031122	cytoplasmic microtubule organization	0.0002732	0.0030247	0.002435161	24
BP	GO:0006271	DNA strand elongation involved in DNA replication	0.0002801	0.0030901	0.002487794	11
BP	GO:0043923	positive regulation by host of viral transcription	0.0002801	0.0030901	0.002487794	11
BP	GO:0030111	regulation of Wnt signaling pathway	0.0002883	0.0031759	0.002556907	112
BP	GO:0006362	transcription elongation from RNA polymerase I promoter	0.0002908	0.0031858	0.002564835	16
BP	GO:1902751	positive regulation of cell cycle G2/M phase transition	0.0002908	0.0031858	0.002564835	16
BP	GO:1904292	regulation of ERAD pathway	0.0002908	0.0031858	0.002564835	16
BP	GO:0043405	regulation of MAP kinase activity	0.0002954	0.0032303	0.002600665	105
BP	GO:0000281	mitotic cytokinesis	0.0003084	0.0033673	0.002711	30
BP	GO:0002218	activation of innate immune response	0.0003109	0.0033879	0.002727593	51
BP	GO:0072659	protein localization to plasma membrane	0.000322	0.0035036	0.002820745	84
BP	GO:0006370	7-methylguanosine mRNA capping	0.0003256	0.0035242	0.002837301	17

BP	GO:0043928	exonucleolytic catabolism of deadenylated mRNA	0.0003256	0.0035242	0.002837301	17
BP	GO:0071985	multivesicular body sorting pathway	0.0003256	0.0035242	0.002837301	17
BP	GO:0051056	regulation of small GTPase mediated signal transduction	0.0003331	0.0035993	0.002897736	105
BP	GO:0032006	regulation of TOR signaling	0.0003385	0.0036505	0.002938973	40
BP	GO:0006469	negative regulation of protein kinase activity	0.0003469	0.0037352	0.003007169	77
BP	GO:0006361	transcription initiation from RNA polymerase I promoter	0.0003527	0.0037914	0.003052425	18
BP	GO:0008334	histone mRNA metabolic process	0.0003584	0.0038318	0.003084992	14
BP	GO:0009299	mRNA transcription	0.0003584	0.0038318	0.003084992	14
BP	GO:0035459	vesicle cargo loading	0.0003584	0.0038318	0.003084992	14
BP	GO:1903828	negative regulation of cellular protein localization	0.000361	0.0038511	0.003100528	42
BP	GO:0043087	regulation of GTPase activity	0.0003614	0.0038511	0.003100528	142
BP	GO:0051851	modulation by host of symbiont process	0.0003655	0.0038887	0.003130753	26
BP	GO:0072595	maintenance of protein localization in organelle	0.0003725	0.0039488	0.00317913	19
BP	GO:1904591	positive regulation of protein import	0.0003725	0.0039488	0.00317913	19
BP	GO:0051310	metaphase plate congression	0.0003767	0.0039799	0.003204174	25
BP	GO:1901799	negative regulation of proteasomal protein catabolic process	0.0003767	0.0039799	0.003204174	25
BP	GO:1903358	regulation of Golgi organization	0.0003788	0.0039956	0.003216829	10
BP	GO:0090503	RNA phosphodiester bond hydrolysis, exonucleolytic	0.0003854	0.0040583	0.003267292	20

BP	GO:0032479	regulation of type I interferon production	0.0003939	0.0041365	0.003330232	46
BP	GO:2000059	negative regulation of ubiquitin-dependent protein catabolic process	0.0003941	0.0041365	0.003330232	22
BP	GO:0032211	negative regulation of telomere maintenance via telomerase	0.0004037	0.0042152	0.003393659	12
BP	GO:0042276	error-prone translesion synthesis	0.0004037	0.0042152	0.003393659	12
BP	GO:0070987	error-free translesion synthesis	0.0004037	0.0042152	0.003393659	12
BP	GO:0034401	chromatin organization involved in regulation of transcription	0.0004046	0.0042175	0.003395477	48
BP	GO:0090263	positive regulation of canonical Wnt signaling pathway	0.000416	0.004329	0.003485233	52
BP	GO:0051123	RNA polymerase II preinitiation complex assembly	0.0004219	0.0043694	0.003517757	15
BP	GO:0060260	regulation of transcription initiation from RNA polymerase II promoter	0.0004219	0.0043694	0.003517757	15
BP	GO:0070861	regulation of protein exit from endoplasmic reticulum	0.0004219	0.0043694	0.003517757	15
BP	GO:1905037	autophagosome organization	0.0004265	0.0044097	0.00355022	37
BP	GO:0042274	ribosomal small subunit biogenesis	0.0004557	0.0047031	0.003786402	28
BP	GO:0000045	autophagosome assembly	0.0004599	0.004739	0.00381531	36
BP	GO:0036257	multivesicular body organization	0.0004735	0.0048465	0.003901902	16
BP	GO:0045648	positive regulation of erythrocyte differentiation	0.0004735	0.0048465	0.003901902	16
BP	GO:1902186	regulation of viral release from host cell	0.0004735	0.0048465	0.003901902	16
BP	GO:1902895	positive regulation of pri-miRNA transcription by RNA polymerase II	0.0004735	0.0048465	0.003901902	16
BP	GO:0043620	regulation of DNA-templated transcription in response to stress	0.0004819	0.0049251	0.003965154	46

BP	GO:0045185	maintenance of protein location	0.0004954	0.0050543	0.004069168	35
BP	GO:0120034	positive regulation of plasma membrane bounded cell projection assembly	0.0004981	0.0050734	0.004084589	38
BP	GO:0001833	inner cell mass cell proliferation	0.0005013	0.0050819	0.004091386	9
BP	GO:0008298	intracellular mRNA localization	0.0005013	0.0050819	0.004091386	9
BP	GO:0045799	positive regulation of chromatin assembly or disassembly	0.0005013	0.0050819	0.004091386	9
BP	GO:0071902	positive regulation of protein serine/threonine kinase activity	0.0005033	0.0050938	0.004100945	103
BP	GO:0043666	regulation of phosphoprotein phosphatase activity	0.0005094	0.0051467	0.004143549	43
BP	GO:2001233	regulation of apoptotic signaling pathway	0.0005121	0.0051658	0.004158908	122
BP	GO:2000637	positive regulation of gene silencing by miRNA	0.0005169	0.0052058	0.004191124	13
BP	GO:0046578	regulation of Ras protein signal transduction	0.0005318	0.0053471	0.004304885	77
BP	GO:0042306	regulation of protein import into nucleus	0.0005365	0.0053691	0.004322609	24
BP	GO:0048016	inositol phosphate-mediated signaling	0.0005365	0.0053691	0.004322609	24
BP	GO:0002244	hematopoietic progenitor cell differentiation	0.0005372	0.0053691	0.004322609	57
BP	GO:0060828	regulation of canonical Wnt signaling pathway	0.0005374	0.0053691	0.004322609	90
BP	GO:0000289	nuclear-transcribed mRNA poly(A) tail shortening	0.0005405	0.0053736	0.004326213	18
BP	GO:0006296	nucleotide-excision repair, DNA incision, 5'-to lesion	0.0005405	0.0053736	0.004326213	18
BP	GO:2000142	regulation of DNA-templated transcription, initiation	0.0005405	0.0053736	0.004326213	18
BP	GO:0034250	positive regulation of cellular amide metabolic process	0.0005469	0.0054294	0.004371131	53

BP	GO:0006757	ATP generation from ADP	0.0005547	0.0054966	0.004425301	42
BP	GO:2000045	regulation of G1/S transition of mitotic cell cycle	0.0005555	0.0054966	0.004425301	62
BP	GO:0031497	chromatin assembly	0.0005585	0.0055176	0.004442167	67
BP	GO:0060070	canonical Wnt signaling pathway	0.0005652	0.0055664	0.004481491	103
BP	GO:0034198	cellular response to amino acid starvation	0.0005662	0.0055664	0.004481491	20
BP	GO:0051293	establishment of spindle localization	0.0005662	0.0055664	0.004481491	20
BP	GO:0031648	protein destabilization	0.000567	0.0055664	0.004481491	21
BP	GO:0006607	NLS-bearing protein import into nucleus	0.0005707	0.0055939	0.004503642	11
BP	GO:0010923	negative regulation of phosphatase activity	0.000575	0.0056272	0.00453042	39
BP	GO:0042158	lipoprotein biosynthetic process	0.0005827	0.0056844	0.004576458	36
BP	GO:0044728	DNA methylation or demethylation	0.0005827	0.0056844	0.004576458	36
BP	GO:0045931	positive regulation of mitotic cell cycle	0.0005889	0.0057359	0.004617948	56
BP	GO:0045862	positive regulation of proteolysis	0.0005901	0.005739	0.004620438	109
BP	GO:1903900	regulation of viral life cycle	0.0005991	0.0058175	0.004683643	52
BP	GO:0045739	positive regulation of DNA repair	0.0006406	0.0061324	0.004937154	27
BP	GO:0000478	endonucleolytic cleavage involved in rRNA processing	0.0006408	0.0061324	0.004937154	8
BP	GO:0000479	endonucleolytic cleavage of tricistronic rRNA transcript (SSU-rRNA, 5.8S rRNA, LSU-rRNA)	0.0006408	0.0061324	0.004937154	8
BP	GO:0002328	pro-B cell differentiation	0.0006408	0.0061324	0.004937154	8

BP	GO:0031442	positive regulation of mRNA 3'-end processing	0.0006408	0.0061324	0.004937154	8
BP	GO:0042023	DNA endoreduplication	0.0006408	0.0061324	0.004937154	8
BP	GO:0070933	histone H4 deacetylation	0.0006408	0.0061324	0.004937154	8
BP	GO:1900112	regulation of histone H3-K9 trimethylation	0.0006408	0.0061324	0.004937154	8
BP	GO:1905214	regulation of RNA binding	0.0006408	0.0061324	0.004937154	8
BP	GO:0031929	TOR signaling	0.0006414	0.0061324	0.004937154	45
BP	GO:0006165	nucleoside diphosphate phosphorylation	0.0006513	0.0062173	0.005005509	47
BP	GO:0050821	protein stabilization	0.0006683	0.0063707	0.005129	60
BP	GO:0071482	cellular response to light stimulus	0.0007122	0.0067787	0.005457501	46
BP	GO:0031498	chromatin disassembly	0.0007455	0.0070631	0.005686468	12
BP	GO:0046827	positive regulation of protein export from nucleus	0.0007455	0.0070631	0.005686468	12
BP	GO:0070920	regulation of production of small RNA involved in gene silencing by RNA	0.0007455	0.0070631	0.005686468	12
BP	GO:1903051	negative regulation of proteolysis involved in cellular protein catabolic process	0.0007513	0.007107	0.005721829	29
BP	GO:1990778	protein localization to cell periphery	0.0007807	0.0073736	0.005936469	98
BP	GO:0000462	maturation of SSU-rRNA from tricistronic rRNA transcript (SSU-rRNA, 5.8S rRNA, LSU-rRNA)	0.0007852	0.007394	0.005952893	17
BP	GO:0051294	establishment of spindle orientation	0.0007852	0.007394	0.005952893	17
BP	GO:0034975	protein folding in endoplasmic reticulum	0.0008021	0.0075194	0.006053799	10
BP	GO:0043981	histone H4-K5 acetylation	0.0008021	0.0075194	0.006053799	10

BP	GO:0043 982	histone H4-K8 acetylation	0.0008 021	0.0075 194	0.006053 799	10
BP	GO:0010 822	positive regulation of mitochondrion organization	0.0008 355	0.0078 089	0.006286 872	42
BP	GO:1900 180	regulation of protein localization to nucleus	0.0008 355	0.0078 089	0.006286 872	42
BP	GO:0007 032	endosome organization	0.0008 481	0.0079 146	0.006372 014	31
BP	GO:0033 119	negative regulation of RNA splicing	0.0008 917	0.0082 964	0.006679 348	13
BP	GO:0060 148	positive regulation of posttranscriptional gene silencing	0.0008 917	0.0082 964	0.006679 348	13
BP	GO:0046 785	microtubule polymerization	0.0009 107	0.0084 498	0.006802 865	30
BP	GO:0006 096	glycolytic process	0.0009 122	0.0084 498	0.006802 865	41
BP	GO:0035 821	modulation of process of other organism	0.0009 122	0.0084 498	0.006802 865	41
BP	GO:1901 653	cellular response to peptide	0.0009 241	0.0085 476	0.006881 638	115
BP	GO:0033 673	negative regulation of kinase activity	0.0009 385	0.0086 68	0.006978 524	81
BP	GO:0045 727	positive regulation of translation	0.0009 418	0.0086 855	0.006992 651	45
BP	GO:0046 939	nucleotide phosphorylation	0.0009 45	0.0087 022	0.007006 042	47
BP	GO:0090 342	regulation of cell aging	0.0009 497	0.0087 319	0.007030 016	25
BP	GO:0060 491	regulation of cell projection assembly	0.0010 054	0.0092 049	0.007410 808	59
BP	GO:0000 460	maturation of 5.8S rRNA	0.0010 055	0.0092 049	0.007410 808	14
BP	GO:0003 148	outflow tract septum morphogenesis	0.0010 055	0.0092 049	0.007410 808	14
BP	GO:0043 547	positive regulation of GTPase activity	0.0010 085	0.0092 189	0.007422 049	120

BP	GO:0046474	glycerophospholipid biosynthetic process	0.0010113	0.0092304	0.007431295	70
BP	GO:0006109	regulation of carbohydrate metabolic process	0.0010489	0.0095595	0.00769626	65
BP	GO:0006337	nucleosome disassembly	0.0010748	0.0097526	0.007851774	11
BP	GO:0016075	rRNA catabolic process	0.0010748	0.0097526	0.007851774	11
BP	GO:0045947	negative regulation of translational initiation	0.0010748	0.0097526	0.007851774	11
BP	GO:0033045	regulation of sister chromatid segregation	0.0010841	0.0097878	0.007880074	31
BP	GO:0036258	multivesicular body assembly	0.0010881	0.0097878	0.007880074	15
BP	GO:0044818	mitotic G2/M transition checkpoint	0.0010881	0.0097878	0.007880074	15
BP	GO:0045948	positive regulation of translational initiation	0.0010881	0.0097878	0.007880074	15
BP	GO:0061842	microtubule organizing center localization	0.0010881	0.0097878	0.007880074	15
BP	GO:0071480	cellular response to gamma radiation	0.0010881	0.0097878	0.007880074	15
BP	GO:0010457	centriole-centriole cohesion	0.0011174	0.0099794	0.008034309	9
BP	GO:0033169	histone H3-K9 demethylation	0.0011174	0.0099794	0.008034309	9
BP	GO:0048308	organelle inheritance	0.0011174	0.0099794	0.008034309	9
BP	GO:0048313	Golgi inheritance	0.0011174	0.0099794	0.008034309	9
BP	GO:0090069	regulation of ribosome biogenesis	0.0011174	0.0099794	0.008034309	9
BP	GO:0045637	regulation of myeloid cell differentiation	0.0011244	0.0100191	0.008066289	79
BP	GO:0051972	regulation of telomerase activity	0.001125	0.0100191	0.008066289	21

BP	GO:0043618	regulation of transcription from RNA polymerase II promoter in response to stress	0.0011284	0.0100344	0.008078646	43
BP	GO:0030010	establishment of cell polarity	0.0011317	0.0100503	0.008091406	47
BP	GO:0007044	cell-substrate junction assembly	0.0011412	0.0101005	0.008131868	36
BP	GO:0150115	cell-substrate junction organization	0.0011412	0.0101005	0.008131868	36
BP	GO:0070536	protein K63-linked deubiquitination	0.0011423	0.0101005	0.008131868	16
BP	GO:0033683	nucleotide-excision repair, DNA incision	0.0011817	0.0104194	0.008388604	18
BP	GO:0042769	DNA damage response, detection of DNA damage	0.0011817	0.0104194	0.008388604	18
BP	GO:0036498	IRE1-mediated unfolded protein response	0.0011884	0.0104494	0.008412765	26
BP	GO:0048278	vesicle docking	0.0011884	0.0104494	0.008412765	26
BP	GO:0042177	negative regulation of protein catabolic process	0.0012407	0.0108936	0.008770382	46
BP	GO:0034249	negative regulation of cellular amide metabolic process	0.0012646	0.0110877	0.008926638	72
BP	GO:0120032	regulation of plasma membrane bounded cell projection assembly	0.0012868	0.0112671	0.009071055	58
BP	GO:0016331	morphogenesis of embryonic epithelium	0.0013088	0.0114436	0.009213144	51
BP	GO:0051058	negative regulation of small GTPase mediated signal transduction	0.0013368	0.0116714	0.009396569	24
BP	GO:0097549	chromatin organization involved in negative regulation of transcription	0.0013612	0.0118681	0.009554909	43
CC	GO:0016607	nuclear speck	4.89E-37	3.54E-34	2.40E-34	204
CC	GO:0005681	spliceosomal complex	3.06E-31	1.11E-28	7.50E-29	115
CC	GO:0005667	transcription regulator complex	6.00E-25	1.45E-22	9.82E-23	187

CC	GO:0061695	transferase complex, transferring phosphorus-containing groups	1.63E-24	2.85E-22	1.93E-22	133
CC	GO:0000151	ubiquitin ligase complex	1.97E-24	2.85E-22	1.93E-22	141
CC	GO:0098687	chromosomal region	2.91E-24	3.52E-22	2.38E-22	163
CC	GO:0005635	nuclear envelope	2.42E-20	2.40E-18	1.62E-18	192
CC	GO:0071013	catalytic step 2 spliceosome	2.65E-20	2.40E-18	1.62E-18	60
CC	GO:0034708	methyltransferase complex	3.37E-20	2.71E-18	1.84E-18	71
CC	GO:0005819	spindle	5.61E-20	4.06E-18	2.75E-18	154
CC	GO:0000790	nuclear chromatin	8.06E-20	5.30E-18	3.59E-18	163
CC	GO:0031248	protein acetyltransferase complex	1.91E-17	1.06E-15	7.19E-16	60
CC	GO:1902493	acetyltransferase complex	1.91E-17	1.06E-15	7.19E-16	60
CC	GO:0005643	nuclear pore	4.70E-17	2.43E-15	1.65E-15	57
CC	GO:0000775	chromosome, centromeric region	2.42E-16	1.17E-14	7.90E-15	95
CC	GO:0055029	nuclear DNA-directed RNA polymerase complex	4.39E-16	1.91E-14	1.30E-14	63
CC	GO:0090575	RNA polymerase II transcription regulator complex	4.50E-16	1.91E-14	1.30E-14	85
CC	GO:0005684	U2-type spliceosomal complex	5.92E-16	2.38E-14	1.61E-14	56
CC	GO:0000428	DNA-directed RNA polymerase complex	8.24E-16	3.14E-14	2.13E-14	63
CC	GO:0030880	RNA polymerase complex	1.96E-15	7.09E-14	4.80E-14	64
CC	GO:0000123	histone acetyltransferase complex	3.13E-15	1.06E-13	7.18E-14	53

CC	GO:0035770	ribonucleoprotein granule	3.22E-15	1.06E-13	7.18E-14	103
CC	GO:0035097	histone methyltransferase complex	6.38E-15	2.01E-13	1.36E-13	53
CC	GO:0000118	histone deacetylase complex	1.17E-14	3.53E-13	2.39E-13	46
CC	GO:0016605	PML body	4.25E-14	1.23E-12	8.34E-13	57
CC	GO:0031965	nuclear membrane	2.87E-13	8.00E-12	5.42E-12	122
CC	GO:0000922	spindle pole	3.38E-13	9.07E-12	6.14E-12	79
CC	GO:0036464	cytoplasmic ribonucleoprotein granule	3.65E-13	9.43E-12	6.39E-12	95
CC	GO:0005657	replication fork	4.62E-13	1.15E-11	7.82E-12	44
CC	GO:1904949	ATPase complex	5.07E-13	1.22E-11	8.29E-12	48
CC	GO:0070603	SWI/SNF superfamily-type complex	9.90E-13	2.31E-11	1.57E-11	46
CC	GO:0034399	nuclear periphery	2.20E-12	4.98E-11	3.38E-11	66
CC	GO:0000776	kinetochore	1.26E-11	2.76E-10	1.87E-10	66
CC	GO:0018995	host cellular component	1.95E-11	4.04E-10	2.74E-10	43
CC	GO:0043657	host cell	1.95E-11	4.04E-10	2.74E-10	43
CC	GO:0000781	chromosome, telomeric region	3.25E-11	6.37E-10	4.31E-10	73
CC	GO:0031461	cullin-RING ubiquitin ligase complex	3.25E-11	6.37E-10	4.31E-10	73
CC	GO:0071007	U2-type catalytic step 2 spliceosome	3.60E-11	6.86E-10	4.65E-10	24
CC	GO:0016363	nuclear matrix	3.72E-11	6.91E-10	4.68E-10	56

CC	GO:0016591	RNA polymerase II, holoenzyme	7.19E-11	1.30E-09	8.81E-10	46
CC	GO:0071011	precatalytic spliceosome	1.24E-10	2.20E-09	1.49E-09	33
CC	GO:0071005	U2-type precatalytic spliceosome	3.62E-10	6.24E-09	4.23E-09	32
CC	GO:0000784	nuclear chromosome, telomeric region	3.94E-10	6.64E-09	4.50E-09	59
CC	GO:0090734	site of DNA damage	4.42E-10	7.28E-09	4.93E-09	41
CC	GO:1902911	protein kinase complex	4.81E-10	7.74E-09	5.24E-09	54
CC	GO:0072686	mitotic spindle	1.64E-09	2.58E-08	1.75E-08	53
CC	GO:1902554	serine/threonine protein kinase complex	3.68E-09	5.67E-08	3.84E-08	45
CC	GO:0017053	transcription repressor complex	3.83E-09	5.77E-08	3.91E-08	41
CC	GO:0008023	transcription elongation factor complex	1.42E-08	2.10E-07	1.42E-07	33
CC	GO:0010494	cytoplasmic stress granule	1.46E-08	2.11E-07	1.43E-07	36
CC	GO:0005814	centriole	1.51E-08	2.14E-07	1.45E-07	61
CC	GO:1902562	H4 histone acetyltransferase complex	1.59E-08	2.22E-07	1.50E-07	27
CC	GO:0019908	nuclear cyclin-dependent protein kinase holoenzyme complex	1.63E-08	2.22E-07	1.51E-07	12
CC	GO:0000793	condensed chromosome	1.67E-08	2.24E-07	1.52E-07	87
CC	GO:0043596	nuclear replication fork	2.18E-08	2.87E-07	1.94E-07	26
CC	GO:0031984	organelle subcompartment	2.43E-08	3.15E-07	2.13E-07	132
CC	GO:0000792	heterochromatin	3.52E-08	4.47E-07	3.03E-07	39

CC	GO:0016592	mediator complex	4.01E-08	5.00E-07	3.39E-07	24
CC	GO:0005925	focal adhesion	8.26E-08	1.01E-06	6.87E-07	137
CC	GO:0005793	endoplasmic reticulum-Golgi intermediate compartment	9.41E-08	1.14E-06	7.69E-07	55
CC	GO:0000777	condensed chromosome kinetochore	1.09E-07	1.30E-06	8.79E-07	48
CC	GO:0030055	cell-substrate junction	1.43E-07	1.65E-06	1.12E-06	138
CC	GO:0000779	condensed chromosome, centromeric region	1.44E-07	1.65E-06	1.12E-06	52
CC	GO:0031519	PcG protein complex	2.23E-07	2.53E-06	1.71E-06	27
CC	GO:0035861	site of double-strand break	2.68E-07	2.98E-06	2.02E-06	31
CC	GO:0098791	Golgi apparatus subcompartment	3.15E-07	3.45E-06	2.34E-06	122
CC	GO:0030496	midbody	4.22E-07	4.56E-06	3.09E-06	68
CC	GO:0030894	replisome	4.80E-07	5.11E-06	3.46E-06	18
CC	GO:0044615	nuclear pore nuclear basket	6.92E-07	7.26E-06	4.92E-06	11
CC	GO:0005669	transcription factor TFIID complex	9.31E-07	9.63E-06	6.53E-06	23
CC	GO:0070822	Sin3-type complex	1.02E-06	1.04E-05	7.01E-06	13
CC	GO:0097431	mitotic spindle pole	1.14E-06	1.14E-05	7.74E-06	18
CC	GO:0000307	cyclin-dependent protein kinase holoenzyme complex	1.20E-06	1.17E-05	7.95E-06	24
CC	GO:1990391	DNA repair complex	1.20E-06	1.17E-05	7.95E-06	24
CC	GO:0001650	fibrillar center	1.50E-06	1.45E-05	9.79E-06	54

CC	GO:0090543	Flemming body	2.51E-06	2.39E-05	1.62E-05	18
CC	GO:0000932	P-body	3.06E-06	2.87E-05	1.95E-05	38
CC	GO:0005798	Golgi-associated vesicle	3.10E-06	2.87E-05	1.95E-05	67
CC	GO:0016580	Sin3 complex	3.58E-06	3.28E-05	2.22E-05	11
CC	GO:0030684	preribosome	4.51E-06	4.08E-05	2.77E-05	35
CC	GO:0043601	nuclear replisome	4.57E-06	4.08E-05	2.77E-05	16
CC	GO:0008287	protein serine/threonine phosphatase complex	6.25E-06	5.45E-05	3.69E-05	24
CC	GO:1903293	phosphatase complex	6.25E-06	5.45E-05	3.69E-05	24
CC	GO:0035267	NuA4 histone acetyltransferase complex	7.79E-06	6.63E-05	4.49E-05	14
CC	GO:0043189	H4/H2A histone acetyltransferase complex	7.79E-06	6.63E-05	4.49E-05	14
CC	GO:0000803	sex chromosome	1.03E-05	8.69E-05	5.88E-05	18
CC	GO:0036064	ciliary basal body	1.10E-05	9.15E-05	6.20E-05	52
CC	GO:0032040	small-subunit processome	1.14E-05	9.38E-05	6.35E-05	21
CC	GO:0031080	nuclear pore outer ring	1.15E-05	9.38E-05	6.35E-05	9
CC	GO:0005874	microtubule	1.79E-05	0.0001443	9.78E-05	130
CC	GO:0000242	pericentriolar material	1.85E-05	0.0001457	9.87E-05	14
CC	GO:0032806	carboxy-terminal domain protein kinase complex	1.85E-05	0.0001457	9.87E-05	14
CC	GO:0005802	trans-Golgi network	1.90E-05	0.0001477	0.000100102	81

CC	GO:0030014	CCR4-NOT complex	3.17E-05	0.0002444	0.000165559	12
CC	GO:0016581	NuRD complex	3.96E-05	0.0002963	0.000200766	11
CC	GO:0090545	CHD-type complex	3.96E-05	0.0002963	0.000200766	11
CC	GO:0005689	U12-type spliceosomal complex	4.01E-05	0.0002963	0.000200766	16
CC	GO:0030137	COPI-coated vesicle	4.01E-05	0.0002963	0.000200766	16
CC	GO:0019005	SCF ubiquitin ligase complex	4.23E-05	0.000309	0.000209357	29
CC	GO:0005795	Golgi stack	4.29E-05	0.0003106	0.000210411	55
CC	GO:0071014	post-mRNA release spliceosomal complex	4.68E-05	0.0003356	0.00022736	10
CC	GO:0035145	exon-exon junction complex	5.56E-05	0.0003949	0.000267563	13
CC	GO:0010008	endosome membrane	5.68E-05	0.0003993	0.000270517	144
CC	GO:0080008	Cul4-RING E3 ubiquitin ligase complex	6.10E-05	0.0004249	0.000287898	18
CC	GO:0070461	SAGA-type complex	7.41E-05	0.0005108	0.000346097	16
CC	GO:0099023	vesicle tethering complex	8.43E-05	0.0005755	0.000389942	29
CC	GO:0016234	inclusion body	8.99E-05	0.0006085	0.000412241	34
CC	GO:0035098	ESC/E(Z) complex	0.0001009	0.0006762	0.000458118	11
CC	GO:0005697	telomerase holoenzyme complex	0.000116	0.0007633	0.000517125	13
CC	GO:0042575	DNA polymerase complex	0.000116	0.0007633	0.000517125	13
CC	GO:0000152	nuclear ubiquitin ligase complex	0.0001275	0.0008319	0.000563643	21

CC	GO:0044665	MLL1/2 complex	0.0001309	0.0008384	0.000568061	16
CC	GO:0071339	MLL1 complex	0.0001309	0.0008384	0.000568061	16
CC	GO:0030135	coated vesicle	0.0001373	0.0008718	0.000590632	92
CC	GO:0031252	cell leading edge	0.0001467	0.0009234	0.00062563	122
CC	GO:0071564	npBAF complex	0.0001619	0.0010103	0.000684474	9
CC	GO:0016514	SWI/SNF complex	0.0001633	0.0010103	0.000684516	12
CC	GO:0005876	spindle microtubule	0.0001821	0.0011172	0.000756938	26
CC	GO:0030027	lamellipodium	0.0002223	0.0013526	0.000916411	65
CC	GO:0035869	ciliary transition zone	0.0002302	0.0013887	0.000940906	28
CC	GO:1905368	peptidase complex	0.0002491	0.0014904	0.001009807	35
CC	GO:0030173	integral component of Golgi membrane	0.0002532	0.0015028	0.001018205	26
CC	GO:0097346	INO80-type complex	0.0002809	0.0016533	0.001120126	14
CC	GO:0033116	endoplasmic reticulum-Golgi intermediate compartment membrane	0.0003902	0.002278	0.001543387	29
CC	GO:0031463	Cul3-RING ubiquitin ligase complex	0.000407	0.0023576	0.001597325	18
CC	GO:0042405	nuclear inclusion body	0.0004207	0.0023985	0.001625034	9
CC	GO:1990023	mitotic spindle midzone	0.0004207	0.0023985	0.001625034	9
CC	GO:0031228	intrinsic component of Golgi membrane	0.0004447	0.0025153	0.001704175	27
CC	GO:1990907	beta-catenin-TCF complex	0.0005466	0.0030675	0.002078278	8

CC	GO:0005720	nuclear heterochromatin	0.0008875	0.0049426	0.003348739	16
CC	GO:0030120	vesicle coat	0.000966	0.005339	0.003617273	24
CC	GO:0035327	transcriptionally active chromatin	0.0011831	0.0064893	0.004396663	13
CC	GO:0030426	growth cone	0.0012359	0.0067279	0.00455829	56
CC	GO:0030117	membrane coat	0.0012689	0.0068052	0.004610683	35
CC	GO:0048475	coated membrane	0.0012689	0.0068052	0.004610683	35
CC	GO:0030686	90S preribosome	0.0013133	0.0068052	0.004610683	15
CC	GO:0000812	Swr1 complex	0.0013159	0.0068052	0.004610683	8
CC	GO:0042555	MCM complex	0.0013159	0.0068052	0.004610683	8
CC	GO:0071006	U2-type catalytic step 1 spliceosome	0.0013159	0.0068052	0.004610683	8
CC	GO:0071012	catalytic step 1 spliceosome	0.0013159	0.0068052	0.004610683	8
CC	GO:0001726	ruffle	0.0014381	0.0073843	0.005002996	56
CC	GO:0005721	pericentric heterochromatin	0.0015659	0.0078732	0.005334278	11
CC	GO:0005849	mRNA cleavage factor complex	0.0015659	0.0078732	0.005334278	11
CC	GO:0030663	COPI-coated vesicle membrane	0.0015659	0.0078732	0.005334278	11
CC	GO:0031010	ISWI-type complex	0.0017936	0.008892	0.006024548	7
CC	GO:0031414	N-terminal protein acetyltransferase complex	0.0017936	0.008892	0.006024548	7
CC	GO:0015030	Cajal body	0.0018054	0.008892	0.006024548	29

CC	GO:0005662	DNA replication factor A complex	0.0018879	0.0091732	0.006215046	9
CC	GO:0005671	Ada2/Gcn5/Ada3 transcription activator complex	0.0018879	0.0091732	0.006215046	9
CC	GO:0034719	SMN-Sm protein complex	0.002325	0.0112222	0.007603307	10
CC	GO:0005637	nuclear inner membrane	0.0023929	0.0114734	0.007773477	23
CC	GO:0030427	site of polarized growth	0.0025669	0.0122266	0.008283812	56
CC	GO:0000930	gamma-tubulin complex	0.0026251	0.0123414	0.008361601	11
CC	GO:0044232	organelle membrane contact site	0.0026251	0.0123414	0.008361601	11
CC	GO:0000974	Prp19 complex	0.0027482	0.0126734	0.008586507	8
CC	GO:0008250	oligosaccharyltransferase complex	0.0027482	0.0126734	0.008586507	8
CC	GO:0016281	eukaryotic translation initiation factor 4F complex	0.0027482	0.0126734	0.008586507	8
CC	GO:0044322	endoplasmic reticulum quality control compartment	0.0028062	0.012859	0.008712239	12
CC	GO:0005769	early endosome	0.0028889	0.013137	0.008900647	101
CC	GO:0034451	centriolar satellite	0.0029032	0.013137	0.008900647	14
MF	GO:0042393	histone binding	9.69E-36	1.04E-32	8.11E-33	131
MF	GO:0003713	transcription coactivator activity	2.18E-24	1.18E-21	9.13E-22	162
MF	GO:0019787	ubiquitin-like protein transferase activity	8.81E-23	3.16E-20	2.46E-20	190
MF	GO:0004842	ubiquitin-protein transferase activity	2.62E-21	7.05E-19	5.48E-19	178
MF	GO:0140297	DNA-binding transcription factor binding	1.95E-19	4.21E-17	3.27E-17	163

MF	GO:0004386	helicase activity	1.07E-17	1.92E-15	1.49E-15	91
MF	GO:0061629	RNA polymerase II-specific DNA-binding transcription factor binding	3.19E-16	4.92E-14	3.82E-14	130
MF	GO:0008094	DNA-dependent ATPase activity	3.63E-15	4.89E-13	3.80E-13	67
MF	GO:0140030	modification-dependent protein binding	2.10E-14	2.52E-12	1.96E-12	78
MF	GO:0003714	transcription corepressor activity	7.96E-14	8.58E-12	6.66E-12	111
MF	GO:0034212	peptide N-acetyltransferase activity	9.07E-14	8.89E-12	6.90E-12	49
MF	GO:0035257	nuclear hormone receptor binding	2.74E-13	2.46E-11	1.91E-11	79
MF	GO:0016887	ATPase activity	1.57E-12	1.30E-10	1.01E-10	172
MF	GO:0061733	peptide-lysine-N-acetyltransferase activity	3.43E-12	2.64E-10	2.05E-10	43
MF	GO:0008080	N-acetyltransferase activity	3.95E-12	2.84E-10	2.20E-10	53
MF	GO:0004402	histone acetyltransferase activity	4.27E-12	2.88E-10	2.24E-10	42
MF	GO:0070491	repressing transcription factor binding	4.54E-12	2.88E-10	2.24E-10	45
MF	GO:0061659	ubiquitin-like protein ligase activity	1.34E-11	8.05E-10	6.25E-10	103
MF	GO:0044389	ubiquitin-like protein ligase binding	2.81E-11	1.59E-09	1.24E-09	128
MF	GO:0140097	catalytic activity, acting on DNA	4.25E-11	2.29E-09	1.78E-09	96
MF	GO:0016407	acetyltransferase activity	5.20E-11	2.67E-09	2.07E-09	59
MF	GO:0004674	protein serine/threonine kinase activity	1.53E-10	7.52E-09	5.83E-09	167
MF	GO:0031625	ubiquitin protein ligase binding	1.61E-10	7.55E-09	5.86E-09	120

MF	GO:0061630	ubiquitin protein ligase activity	2.06E-10	9.27E-09	7.20E-09	97
MF	GO:0003697	single-stranded DNA binding	2.17E-10	9.35E-09	7.26E-09	59
MF	GO:0003678	DNA helicase activity	5.56E-10	2.30E-08	1.79E-08	46
MF	GO:0016410	N-acyltransferase activity	7.32E-10	2.92E-08	2.27E-08	58
MF	GO:0051427	hormone receptor binding	1.14E-09	4.37E-08	3.39E-08	83
MF	GO:0035064	methylated histone binding	1.26E-09	4.53E-08	3.52E-08	39
MF	GO:0140034	methylation-dependent protein binding	1.26E-09	4.53E-08	3.52E-08	39
MF	GO:0140098	catalytic activity, acting on RNA	3.41E-09	1.18E-07	9.20E-08	146
MF	GO:0070577	lysine-acetylated histone binding	4.23E-09	1.38E-07	1.07E-07	17
MF	GO:0140033	acetylation-dependent protein binding	4.23E-09	1.38E-07	1.07E-07	17
MF	GO:0003727	single-stranded RNA binding	1.21E-08	3.85E-07	2.99E-07	49
MF	GO:0019902	phosphatase binding	1.74E-08	5.37E-07	4.16E-07	80
MF	GO:0030374	nuclear receptor transcription coactivator activity	1.82E-08	5.44E-07	4.22E-07	38
MF	GO:0032182	ubiquitin-like protein binding	1.89E-08	5.44E-07	4.22E-07	49
MF	GO:0046332	SMAD binding	1.92E-08	5.44E-07	4.22E-07	43
MF	GO:0042826	histone deacetylase binding	3.12E-08	8.63E-07	6.70E-07	54
MF	GO:0070063	RNA polymerase binding	3.55E-08	9.58E-07	7.44E-07	41
MF	GO:0002039	p53 binding	4.26E-08	1.10E-06	8.50E-07	37

MF	GO:0047485	protein N-terminus binding	4.27E-08	1.10E-06	8.50E-07	53
MF	GO:0017016	Ras GTPase binding	7.33E-08	1.84E-06	1.43E-06	154
MF	GO:0003725	double-stranded RNA binding	7.92E-08	1.94E-06	1.51E-06	40
MF	GO:0003684	damaged DNA binding	9.86E-08	2.36E-06	1.83E-06	36
MF	GO:0031267	small GTPase binding	1.41E-07	3.30E-06	2.56E-06	157
MF	GO:0017056	structural constituent of nuclear pore	2.30E-07	5.14E-06	3.99E-06	20
MF	GO:0050681	androgen receptor binding	2.32E-07	5.14E-06	3.99E-06	27
MF	GO:0001098	basal transcription machinery binding	2.38E-07	5.14E-06	3.99E-06	38
MF	GO:0001099	basal RNA polymerase II transcription machinery binding	2.38E-07	5.14E-06	3.99E-06	38
MF	GO:0001103	RNA polymerase II repressing transcription factor binding	3.07E-07	6.50E-06	5.04E-06	23
MF	GO:0045296	cadherin binding	3.33E-07	6.91E-06	5.37E-06	122
MF	GO:0061650	ubiquitin-like protein conjugating enzyme activity	3.94E-07	8.01E-06	6.22E-06	25
MF	GO:0140142	nucleocytoplasmic carrier activity	4.70E-07	9.38E-06	7.28E-06	21
MF	GO:0061631	ubiquitin conjugating enzyme activity	5.09E-07	9.97E-06	7.74E-06	24
MF	GO:0046966	thyroid hormone receptor binding	5.69E-07	1.09E-05	8.50E-06	20
MF	GO:0019903	protein phosphatase binding	6.06E-07	1.15E-05	8.89E-06	61
MF	GO:0043175	RNA polymerase core enzyme binding	6.94E-07	1.29E-05	1.00E-05	33
MF	GO:0016922	nuclear receptor binding	1.35E-06	2.47E-05	1.92E-05	50

MF	GO:0008022	protein C-terminus binding	1.74E-06	3.12E-05	2.42E-05	75
MF	GO:0019783	ubiquitin-like protein-specific protease activity	1.97E-06	3.49E-05	2.71E-05	54
MF	GO:0003730	mRNA 3'-UTR binding	2.06E-06	3.59E-05	2.79E-05	43
MF	GO:0061608	nuclear import signal receptor activity	2.95E-06	4.99E-05	3.87E-05	15
MF	GO:0035258	steroid hormone receptor binding	2.96E-06	4.99E-05	3.87E-05	43
MF	GO:0008353	RNA polymerase II CTD heptapeptide repeat kinase activity	3.09E-06	5.13E-05	3.98E-05	14
MF	GO:0003724	RNA helicase activity	3.18E-06	5.19E-05	4.03E-05	38
MF	GO:0008175	tRNA methyltransferase activity	4.46E-06	7.17E-05	5.57E-05	21
MF	GO:0008139	nuclear localization sequence binding	5.57E-06	8.83E-05	6.85E-05	17
MF	GO:0043021	ribonucleoprotein complex binding	6.18E-06	9.66E-05	7.50E-05	56
MF	GO:0140296	general transcription initiation factor binding	6.45E-06	9.93E-05	7.71E-05	24
MF	GO:0051219	phosphoprotein binding	7.19E-06	0.0001091	8.47E-05	39
MF	GO:0001046	core promoter sequence-specific DNA binding	8.23E-06	0.0001233	9.57E-05	25
MF	GO:0000993	RNA polymerase II complex binding	8.80E-06	0.0001299	0.000100876	28
MF	GO:0016780	phosphotransferase activity, for other substituted phosphate groups	9.08E-06	0.0001323	0.000102734	14
MF	GO:0042809	vitamin D receptor binding	1.00E-05	0.0001441	0.000111832	12
MF	GO:0016747	transferase activity, transferring acyl groups other than amino-acyl groups	1.33E-05	0.0001893	0.000146938	86
MF	GO:0005048	signal sequence binding	1.38E-05	0.0001937	0.000150392	25

MF	GO:0008757	S-adenosylmethionine-dependent methyltransferase activity	1.46E-05	0.0002015	0.00015645	62
MF	GO:0016796	exonuclease activity, active with either ribo- or deoxyribonucleic acids and producing 5'-phosphomonoesters	1.59E-05	0.0002172	0.000168629	29
MF	GO:0004576	oligosaccharyl transferase activity	2.51E-05	0.0003385	0.00026279	9
MF	GO:0032452	histone demethylase activity	2.58E-05	0.0003391	0.000263228	17
MF	GO:0140457	protein demethylase activity	2.58E-05	0.0003391	0.000263228	17
MF	GO:0032451	demethylase activity	2.81E-05	0.0003607	0.00027997	21
MF	GO:0042162	telomeric DNA binding	2.81E-05	0.0003607	0.00027997	21
MF	GO:1990841	promoter-specific chromatin binding	3.63E-05	0.000461	0.000357867	25
MF	GO:0008187	poly-pyrimidine tract binding	5.17E-05	0.0006475	0.000502643	19
MF	GO:0010485	H4 histone acetyltransferase activity	5.43E-05	0.0006728	0.000522294	14
MF	GO:0008173	RNA methyltransferase activity	6.13E-05	0.000751	0.000582978	31
MF	GO:0036459	thiol-dependent ubiquitinyl hydrolase activity	6.28E-05	0.0007517	0.00058355	47
MF	GO:0101005	ubiquitinyl hydrolase activity	6.28E-05	0.0007517	0.00058355	47
MF	GO:0000979	RNA polymerase II core promoter sequence-specific DNA binding	9.02E-05	0.0010684	0.000829389	19
MF	GO:0043130	ubiquitin binding	9.42E-05	0.0011034	0.000856567	34
MF	GO:0031491	nucleosome binding	9.60E-05	0.0011129	0.000863898	37
MF	GO:0016251	RNA polymerase II general transcription initiation factor activity	0.0001063	0.0012191	0.000946391	10
MF	GO:0043539	protein serine/threonine kinase activator activity	0.0001106	0.0012554	0.000974538	20

MF	GO:0050321	tau-protein kinase activity	0.0001155	0.0012972	0.001007015	14
MF	GO:0003743	translation initiation factor activity	0.0001319	0.0014656	0.001137719	25
MF	GO:0008168	methyltransferase activity	0.0001368	0.0015053	0.001168525	77
MF	GO:0001221	transcription cofactor binding	0.0001492	0.0016082	0.00124842	22
MF	GO:0061980	regulatory RNA binding	0.0001492	0.0016082	0.00124842	22
MF	GO:0008170	N-methyltransferase activity	0.0001632	0.0017416	0.001351983	40
MF	GO:0070717	poly-purine tract binding	0.0001681	0.0017763	0.001378933	17
MF	GO:0016746	transferase activity, transferring acyl groups	0.0001746	0.0018137	0.001407971	91
MF	GO:0004527	exonuclease activity	0.000175	0.0018137	0.001407971	35
MF	GO:0045182	translation regulator activity	0.0001943	0.0019807	0.00153762	54
MF	GO:0004843	thiol-dependent ubiquitin-specific protease activity	0.0001948	0.0019807	0.00153762	43
MF	GO:0003899	DNA-directed 5'-3' RNA polymerase activity	0.0002059	0.0020613	0.001600139	21
MF	GO:0008408	3'-5' exonuclease activity	0.0002066	0.0020613	0.001600139	26
MF	GO:0008234	cysteine-type peptidase activity	0.0002084	0.0020613	0.001600139	67
MF	GO:0031490	chromatin DNA binding	0.0002138	0.0020954	0.001626604	47
MF	GO:0004407	histone deacetylase activity	0.0002292	0.0022259	0.001727952	16
MF	GO:0032183	SUMO binding	0.0002385	0.0022955	0.001781976	11
MF	GO:0016896	exoribonuclease activity, producing 5'-phosphomonoesters	0.0002481	0.0023671	0.001837596	19

MF	GO:0008536	Ran GTPase binding	0.0002844	0.0026896	0.002087948	20
MF	GO:0035198	miRNA binding	0.0002873	0.0026931	0.002090646	17
MF	GO:0035925	mRNA 3'-UTR AU-rich region binding	0.0003122	0.0028767	0.002233138	15
MF	GO:0042974	retinoic acid receptor binding	0.0003122	0.0028767	0.002233138	15
MF	GO:0016706	2-oxoglutarate-dependent dioxygenase activity	0.0003457	0.0031582	0.002451648	22
MF	GO:0033558	protein deacetylase activity	0.0003951	0.0035794	0.002778645	16
MF	GO:0140223	general transcription initiation factor activity	0.0004018	0.0036094	0.002801929	12
MF	GO:0042054	histone methyltransferase activity	0.0004186	0.0037292	0.002894948	26
MF	GO:0003688	DNA replication origin binding	0.0004247	0.0037525	0.002913052	14
MF	GO:0017137	Rab GTPase binding	0.000451	0.0039525	0.003068325	63
MF	GO:0016741	transferase activity, transferring one-carbon groups	0.0004663	0.0040541	0.003147154	78
MF	GO:0051721	protein phosphatase 2A binding	0.0004733	0.004082	0.003168821	17
MF	GO:0008135	translation factor activity, RNA binding	0.0005306	0.0045392	0.003523772	35
MF	GO:0003887	DNA-directed DNA polymerase activity	0.0005435	0.0046137	0.003581571	15
MF	GO:0001085	RNA polymerase II transcription factor binding	0.000589	0.0049606	0.003850895	27
MF	GO:0004532	exoribonuclease activity	0.0006067	0.0050697	0.003935598	19
MF	GO:0016779	nucleotidyltransferase activity	0.0006282	0.0052088	0.004043579	51
MF	GO:0008266	poly(U) RNA binding	0.0006546	0.0053868	0.004181757	16

MF	GO:0018<br>024	histone-lysine N-methyltransferase activity	0.0007<br>036	0.0057<br>464	0.004460<br>85	21
MF	GO:0019<br>003	GDP binding	0.0007<br>63	0.0061<br>545	0.004777<br>683	31
MF	GO:0051<br>018	protein kinase A binding	0.0007<br>65	0.0061<br>545	0.004777<br>683	23
MF	GO:0019<br>208	phosphatase regulator activity	0.0007<br>836	0.0062<br>57	0.004857<br>224	38
MF	GO:0090<br>079	translation regulator activity, nucleic acid binding	0.0008<br>199	0.0064<br>989	0.005045<br>048	42
MF	GO:0032<br>454	histone demethylase activity (H3-K9 specific)	0.0008<br>545	0.0067<br>237	0.005219<br>543	9
MF	GO:0000<br>287	magnesium ion binding	0.0008<br>687	0.0067<br>857	0.005267<br>657	73
MF	GO:0017<br>091	AU-rich element binding	0.0009<br>062	0.0070<br>283	0.005456<br>019	15
MF	GO:0034<br>062	5'-3' RNA polymerase activity	0.0010<br>168	0.0077<br>741	0.006034<br>949	21
MF	GO:0097<br>747	RNA polymerase activity	0.0010<br>168	0.0077<br>741	0.006034<br>949	21
MF	GO:0070<br>412	R-SMAD binding	0.0010<br>288	0.0077<br>895	0.006046<br>943	13
MF	GO:0034<br>713	type I transforming growth factor beta receptor binding	0.0010<br>405	0.0077<br>895	0.006046<br>943	8
MF	GO:0070<br>411	I-SMAD binding	0.0010<br>405	0.0077<br>895	0.006046<br>943	8
MF	GO:0046<br>527	glucosyltransferase activity	0.0010<br>518	0.0078<br>195	0.006070<br>178	11
MF	GO:0001<br>882	nucleoside binding	0.0011<br>263	0.0083<br>157	0.006455<br>438	122
MF	GO:0032<br>549	ribonucleoside binding	0.0011<br>498	0.0084<br>318	0.006545<br>506	120
MF	GO:0000<br>175	3'-5'-exoribonuclease activity	0.0011<br>659	0.0084<br>921	0.006592<br>369	17
MF	GO:0000<br>400	four-way junction DNA binding	0.0014<br>092	0.0101<br>705	0.007895<br>249	10

MF	GO:0019789	SUMO transferase activity	0.0014152	0.0101705	0.007895249	12
MF	GO:0004721	phosphoprotein phosphatase activity	0.0014262	0.0101817	0.007903959	63
MF	GO:0035035	histone acetyltransferase binding	0.0014539	0.0102437	0.007952069	15
MF	GO:0051059	NF-kappaB binding	0.0014539	0.0102437	0.007952069	15
MF	GO:0035091	phosphatidylinositol binding	0.0015627	0.010939	0.008491842	81
MF	GO:0019207	kinase regulator activity	0.0016326	0.0113545	0.00881438	70
MF	GO:0005085	guanyl-nucleotide exchange factor activity	0.0016479	0.0113876	0.008840066	72
MF	GO:0031072	heat shock protein binding	0.0016682	0.0114544	0.008891973	44
MF	GO:0008143	poly(A) binding	0.0017428	0.0118911	0.009230928	13
MF	GO:0019887	protein kinase regulator activity	0.0017735	0.0120241	0.00933418	62
MF	GO:0034061	DNA polymerase activity	0.001856	0.0124503	0.009665039	18
MF	GO:0008276	protein methyltransferase activity	0.0018624	0.0124503	0.009665039	34
MF	GO:0098847	sequence-specific single stranded DNA binding	0.001871	0.0124503	0.009665039	9
MF	GO:0070182	DNA polymerase binding	0.0019455	0.0128667	0.009988339	11

Supplementary Table. 2 The results of significant KEGG pathways and KEGG terms.

ID	Description	pvalue	p.adjust	qvalue	Count
hsa05168	Herpes simplex virus 1 infection	1.05E-30	3.30E-28	2.26E-28	229
hsa04120	Ubiquitin mediated proteolysis	3.30E-16	5.16E-14	3.54E-14	77
hsa03040	Spliceosome	7.95E-16	8.29E-14	5.69E-14	79

hsa04141	Protein processing in endoplasmic reticulum	9.21E-13	7.21E-11	4.95E-11	82
hsa03013	RNA transport	3.20E-12	2.00E-10	1.38E-10	86
hsa03015	mRNA surveillance pathway	8.45E-12	4.41E-10	3.02E-10	54
hsa04110	Cell cycle	2.17E-09	9.71E-08	6.66E-08	59
hsa03022	Basal transcription factors	4.42E-09	1.73E-07	1.19E-07	29
hsa04140	Autophagy - animal	7.46E-08	2.60E-06	1.78E-06	60
hsa05212	Pancreatic cancer	3.21E-07	1.01E-05	6.90E-06	38
hsa05161	Hepatitis B	4.59E-07	1.31E-05	8.96E-06	66
hsa05131	Shigellosis	6.53E-07	1.70E-05	1.17E-05	91
hsa05203	Viral carcinogenesis	9.27E-07	2.23E-05	1.53E-05	78
hsa04218	Cellular senescence	1.20E-06	2.68E-05	1.84E-05	63
hsa05166	Human T-cell leukemia virus 1 infection	2.70E-06	5.64E-05	3.87E-05	81
hsa05135	Yersinia infection	2.98E-06	5.83E-05	4.00E-05	56
hsa04068	FoxO signaling pathway	3.30E-06	6.07E-05	4.16E-05	54
hsa05130	Pathogenic Escherichia coli infection	3.79E-06	6.58E-05	4.52E-05	74
hsa05132	Salmonella infection	4.56E-06	7.51E-05	5.15E-05	89
hsa03030	DNA replication	6.40E-06	9.84E-05	6.75E-05	21
hsa05211	Renal cell carcinoma	6.60E-06	9.84E-05	6.75E-05	33
hsa03420	Nucleotide excision repair	8.25E-06	0.000117341	8.05E-05	25
hsa04722	Neurotrophin signaling pathway	9.63E-06	0.000125887	8.64E-05	49
hsa04350	TGF-beta signaling pathway	9.65E-06	0.000125887	8.64E-05	41
hsa05215	Prostate cancer	2.42E-05	0.000302593	0.000207597	41
hsa03018	RNA degradation	2.85E-05	0.00033363	0.000228891	35
hsa05220	Chronic myeloid leukemia	2.88E-05	0.00033363	0.000228891	34
hsa00510	N-Glycan biosynthesis	3.34E-05	0.000373409	0.000256181	25
hsa04211	Longevity regulating pathway	3.63E-05	0.000378331	0.000259558	38
hsa05235	PD-L1 expression and PD-1 checkpoint pathway in cancer	3.63E-05	0.000378331	0.000259558	38
hsa04330	Notch signaling pathway	3.90E-05	0.000394185	0.000270435	28
hsa04144	Endocytosis	8.57E-05	0.000837959	0.000574891	85
hsa05210	Colorectal cancer	9.49E-05	0.000900071	0.000617503	36
hsa04520	Adherens junction	0.000112354	0.001034322	0.000709607	31
hsa03430	Mismatch repair	0.000123508	0.001104515	0.000757764	14
hsa04710	Circadian rhythm	0.00014262	0.001208417	0.000829047	17
hsa05160	Hepatitis C	0.000142848	0.001208417	0.000829047	57

hsa04919	Thyroid hormone signaling pathway	0.000181292	0.001493273	0.001024475	46
hsa04010	MAPK signaling pathway	0.000203889	0.001636341	0.001122629	95
hsa03410	Base excision repair	0.000386142	0.003021564	0.002072974	17
hsa04012	ErbB signaling pathway	0.000412528	0.003149299	0.002160609	34
hsa05222	Small cell lung cancer	0.000466196	0.003474273	0.00238356	36
hsa04114	Oocyte meiosis	0.000484081	0.003523661	0.002417443	47
hsa05165	Human papillomavirus infection	0.000529669	0.003767871	0.002584986	103
hsa03450	Non-homologous end-joining	0.000560415	0.00383721	0.002632557	9
hsa05163	Human cytomegalovirus infection	0.000563935	0.00383721	0.002632557	74
hsa03060	Protein export	0.000595684	0.003967001	0.002721602	13
hsa04210	Apoptosis	0.000956054	0.00619349	0.004249107	48
hsa04668	TNF signaling pathway	0.000969588	0.00619349	0.004249107	41
hsa04510	Focal adhesion	0.001147538	0.007183586	0.004928373	66
hsa03008	Ribosome biogenesis in eukaryotes	0.001288577	0.007908326	0.005425588	40
hsa00513	Various types of N-glycan biosynthesis	0.001395593	0.008400396	0.005763177	18
hsa04137	Mitophagy - animal	0.001783185	0.010530886	0.007224822	27
hsa03020	RNA polymerase	0.001927324	0.010987481	0.007538073	15
hsa04071	Sphingolipid signaling pathway	0.001930708	0.010987481	0.007538073	42
hsa05169	Epstein-Barr virus infection	0.002197142	0.012280455	0.008425131	65
hsa01521	EGFR tyrosine kinase inhibitor resistance	0.002329375	0.012791129	0.008775484	30
hsa04659	Th17 cell differentiation	0.002732399	0.014549313	0.009981704	38
hsa04392	Hippo signaling pathway - multiple species	0.002788025	0.014549313	0.009981704	14
hsa03440	Homologous recombination	0.002789006	0.014549313	0.009981704	18
